# Development of a portfolio of learning for postgraduate family medicine training in South Africa: a Delphi study

**DOI:** 10.1186/1471-2296-13-11

**Published:** 2012-03-03

**Authors:** Louis Jenkins, Bob Mash, Anselme Derese

**Affiliations:** 1 University of Stellenbosch, Division of Family Medicine and Primary Care, George Training Complex, George, South Africa; 2 University of Stellenbosch, Division of Family Medicine and Primary Care, Tygerberg, South Africa; 3 Centre for Education Development, Faculty of Medicine and Health Sciences, Ghent University, Ghent, Belgium

## Abstract

**Background:**

Within the 52 health districts in South Africa, the family physician is seen as the clinical leader within a multi-professional district health team. Family physicians must be competent to meet 90% of the health needs of the communities in their districts. The eight university departments of Family Medicine have identified five unit standards, broken down into 85 training outcomes, for postgraduate training. The family medicine registrar must prove at the end of training that all the required training outcomes have been attained. District health managers must be assured that the family physician is competent to deliver the expected service. The Colleges of Medicine of South Africa (CMSA) require a portfolio to be submitted as part of the uniform assessment of all registrars applying to write the national fellowship examinations. This study aimed to achieve a consensus on the contents and principles of the first national portfolio for use in family medicine training in South Africa.

**Methods:**

A workshop held at the WONCA Africa Regional Conference in 2009 explored the purpose and broad contents of the portfolio. The 85 training outcomes, ideas from the WONCA workshop, the literature, and existing portfolios in the various universities were used to develop a questionnaire that was tested for content validity by a panel of 31 experts in family medicine in South Africa, via the Delphi technique in four rounds. Eighty five content items (national learning outcomes) and 27 principles were tested. Consensus was defined as 70% agreement. For those items that the panel thought should be included, they were also asked how to provide evidence for the specific item in the portfolio, and how to assess that evidence.

**Results:**

Consensus was reached on 61 of the 85 national learning outcomes. The panel recommended that 50 be assessed by the portfolio and 11 should not be. No consensus could be reached on the remaining 24 outcomes and these were also omitted from the portfolio. The panel recommended that various types of evidence be included in the portfolio. The panel supported 26 of the 27 principles, but could not reach consensus on whether the portfolio should reflect on the relationship between the supervisor and registrar.

**Conclusion:**

A portfolio was developed and distributed to the eight departments of Family Medicine in South Africa, and the CMSA, to be further tested in implementation.

## Background

The National Health Act of South Africa (Act 61 of 2003) identifies the District Health System as the context for 90% of all state health delivery [1]. Within each of the 52 health districts in the country, the family physician is recognized as the person who is primarily responsible for clinical governance. The family physician is seen as the clinical leader of a multi-professional district health team (including nurses, doctors, allied health professions, pharmacists, radiographers, home based carers, and managers). It is expected that family physicians will be competent to meet 90% of the health needs of the communities in their designated districts. The family medicine registrar should be able to prove, at the end of his/her training that all the required training outcomes have been attained. District health managers must be assured that the family physician is competent to deliver the expected service.

The Academy of Family Physicians in South Africa has identified five national unit standards for Family Medicine training [2]. For each unit standard specific training outcomes were identified for the discipline [2]. These training outcomes were revised and updated in 2010. From each of the training outcomes in unit standard two a list of 214 core clinical skills and a number of elective clinical skills were also identified as what is expected from a family physician [3,4].

The five national unit standards for Family Medicine training in South Africa

**1**. Effectively manage him/herself, his/her team and his/her practice, in any sector, with visionary leadership and self-awareness, in order to ensure the provision of high-quality, evidence-based care.

**2**. Evaluate and manage patients with both undifferentiated and more specific problems cost-effectively according to the bio-psycho-social approach

**3**. Facilitate the health and quality of life of the family and community.

**4**. Facilitate the learning of others regarding the discipline of family medicine, primary health care, and other health-related matters

**5**. Conduct all aspects of health care in an ethical and professional manner

Family Medicine was recognized as a specialty in 2007 and all eight departments in the country have established postgraduate training programmes. The departments have various ways of assessing postgraduate outcomes, which include oral examination, written papers and objective structured clinical examinations, simulated consultations and research assignments. These assessments focus on the registrars' knowledge, decision-making skills, communication skills, professional values and practical skills. However the outcomes are often assessed in a deconstructed manner under artificial examination conditions, which does not sufficiently appreciate the complexity of medical practice, where the doctor need to integrate the patient, clinical findings, the context, and him/herself, among many factors. There exists a need to assess competency of family medicine registrars in a more integrated way, looking at their various skills in the context of the workplace (real world). It is clear that some competencies can only be fully assessed in clinical practice [5].

The Colleges of Medicine of South Africa, including the College of Family Physicians, are developing unitary exit examinations for specialist training. Every postgraduate student presenting for the national examination now has to produce a portfolio of learning [6,7]. This portfolio forms part of the summative assessment of the college fellowship examinations.

This development is in line with international interest on in-service assessments, workplace-based competencies, and the use of portfolios in the arts, architecture, teaching and health [8-13]. A learning portfolio that is correctly used can reflect a detailed picture of the registrar's experience and learning in clinical practice over a period of time [14]. The use of portfolios has been shown to be practical and reliable in assessment of undergraduate medical education [15,16]. Apart from use in assessment, it can also function as a reflective journal, a personal development plan, and a skills map. It should stimulate reflective thinking and foster ongoing learning [17]. The possible content and types of evidence for a postgraduate portfolio have been described in the Netherlands [18] and Australia [19]. Thistlethwaite explored the reasons, merits and nature of portfolios in medical education, emphasizing the difference between logbooks (which is a list of required activities or skills to be ticked off) and portfolios (which require evidence that learning has taken place) [19].

In contrast with conventional forms of assessment, which test what registrars know (e.g. multiple choice questions) or what they can show in a simulated environment (e.g. OSCE), the portfolio should provide evidence of whether they can actually perform a skill or competency in a more realistic context (district health system, primary health care), on a more continuous basis (day-to-day clinical practice), finally improving health care in the communities served [20].

The context of learning and the role of a mentor significantly impacts on the learning of a registrar. As opposed to the deconstructed psychometrically-based assessment of learning, a portfolio correctly completed and assessed within the context of everyday work, with an appropriate mentor, increases the validity of learning [5].

Life-long learning in the uncontrolled workplace context, with a commitment to continuously reflect on practice, needs a new way of thinking and of assessing such learning [21-23].

Reflection is a key concept in portfolio learning [24]. Becoming a reflective practitioner requires a huge mind shift in the family medicine registrar and the mentor [23]. The rewards include a greater understanding of self and situations, which will inform future action.

The Association of Medical Education in Europe (AMEE) has raised some critical factors regarding the use of a learning portfolio [25]. These include the need for clear goals, combining summative and formative assessment methods, the importance of a mentor, prioritizing time, and allowing flexibility.

A collaborative workshop held at the WONCA Sub-Saharan Africa Regional Conference in 2009 started the process of designing a national portfolio. The workshop was used to generate and prioritize ideas from the participants that related to the contents and principles of the portfolio. This study aimed to take these ideas forward and to achieve consensus on the content and principles for designing the first national portfolio of learning for family medicine postgraduate training in South Africa.

## Methods

### Study design

The Delphi technique was chosen because this is an ideal method to reach consensus, in this case on the contents and principles of the portfolio. "The Delphi technique is a method of collecting expert opinion on a particular research question. It is based on the premise that pooled intelligence enhances individual judgement and captures the collective opinion of a group of experts without being physically assembled. The conventional Delphi uses a series of questionnaires to generate expert opinion in an anonymous fashion and takes place over a series of rounds." [26] This study involved 4 rounds of questionnaires to an expert panel over a period of one year.

### Participant selection

Ninety three experts in family medicine were invited to join the panel from the following three categories:

A. Supervisors who were responsible for training family medicine registrars

B. Family physicians, including those in academia, who were participating in or managing family medicine training programmes

C. Senior family medicine registrars or recently qualified family physicians

Invitations were also intended to produce a reasonably even representation across all eight universities.

Participants were identified through the heads of the eight departments of family medicine. Out of the 93 people approached 31 gave consent and agreed to participate in the expert panel as shown in Table 1. For every round, the questionnaire was sent to all 31 participants.

**Table 1:** Participants in the Delphi expert panel

	Stellenbosch	Cape Town	Natal	Witwatersrand	Pretoria	Limpopo	Free State	Walter Sisulu	Total
Asked	15	14	12	12	8	12	11	9	93

Consented									

Category A	2	1	1	3	2	1	4	1	15

Category B	1	2	1	2	1	1	2	0	10

Category C	2	0	3	0	0	0	1	0	6

Total	5	3	5	5	3	2	7	1	31

Before the Delphi process began consensus was defined as 70% or more of the group agreeing on an answer and items on which consensus were achieved were removed from subsequent rounds.

#### Round 1

The initial questionnaire (see additional file 1), developed by the principal author, with modifications by the two other authors, asked about the content of the portfolio. Questions were based on the national training outcomes for family medicine as well as the contents of various existing portfolios or logbooks used by individual departments, and ideas generated by the WONCA workshop. The panel was asked to rate each of the 85 content items in the draft portfolio as follows:

A: "must be included for assessment in the portfolio",

B: "should be left out ~ can be assessed better in another way", or

C: "would be good to include, but not sure how to assess".

For those items that they thought should be included panel members were asked to respond to the following open questions with a written response:

1. How to provide evidence for the specific item in the portfolio (e.g. direct observation and assessment by supervisor, written assignment)

2. How to assess the evidence provided (e.g. Likert scale, grade, global rating)

Questions on 27 key principles were derived from the WONCA workshop and the literature. The panel was asked to comment on whether they agreed or disagreed with the principles pertaining to the learning portfolio. If they disagreed, they were asked to comment on why they would reject this principle. They were also asked to add any other principles that they thought should be included.

In each round respondents were also asked to give qualitative feedback on the questions, to suggest new questions or to modify existing ones.

Twenty nine participants returned a completed questionnaire from round 1.

#### Round 2

Items on which there were no consensus, and new items, were presented to the panel in round 2, together with the results and anonymous feedback from the panel's opinion from round 1. During round 2 the participants were asked to select one of three options:

A. "Should primarily be assessed by portfolio."

B. "Should primarily be assessed by other means."

C. "Needs rephrasing. Please rephrase it as you would see it."

Twenty seven participants returned a completed questionnaire from round 2.

#### Round 3

The panel was given anonymous feedback on the voting from round 2. The 16 items where participants did not reach consensus in round 2, were rephrased and the panel asked to vote whether each item should be primarily assessed in the portfolio or primarily be assessed by other means.

Twenty three participants returned a questionnaire from round 3. Consensus was reached on 8 items, and no consensus was reached on 8 items.

During this time the family medicine departments revised the national training outcomes. This resulted in a change of wording or combination of some of the items tested in the previous 3 Delphi rounds, resulting in 29 new or revised outcomes. It was decided to ask the panel whether these outcomes (items) should be included for assessment in the portfolio or not in a 4^th ^Delphi round.

#### Round 4

Twenty three participants returned a questionnaire from round 4. Consensus was reached on 13 of the 29 items. No final consensus could be reached on the remaining 16 items and it was decided that this would be interpreted as a lack of sufficient support to include these in the portfolio.

## Results

### Learning outcomes

Overall the panel recommended that 50 of the 85 national learning outcomes should be assessed by the portfolio and 11 should not be. No consensus could be reached on the remaining 24 outcomes and these were also omitted from the portfolio.

#### Learning outcomes to be assessed in the portfolio (50)

Effectively manage him/herself, his/her team and his/her practice, in any sector, with visionary leadership and self-awareness, in order to ensure the provision of high-quality, evidence-based care.

1. Addressing his/her personal learning needs continually by assessing needs and participating in an appropriate programme of learning.

2. Demonstrating growth and learning in response to identified needs

3. Demonstrating willingness to seek help when necessary

4. Describing activities to enhance self-growth and development

5. Demonstrating ability to develop his/her own capacity

6. Planning, implementing and maintaining information- and record-keeping systems.

7. Demonstrating the ability to plan and conduct a practice audit

8. Implementing ongoing quality improvement activities

9. Critically reviewing research articles and applying the evidence in practice

10. Demonstrating the implementation of research and literature review findings in the management of problems in practice by, for instance, developing protocols for the practice

11. Adapting and implementing appropriate local, national and international clinical guidelines

12. Engaging in monitoring and evaluation to ensure high quality care

13. Implementing rational prescribing and diagnostic testing

14. Communicating and collaborating effectively with members of the health care team and peers

Evaluate and manage patients with both undifferentiated and more specific problems cost-effectively according to the bio-psycho-social approach

15. Taking a relevant history in a patient-centred manner, including exploration of the patient's illness experiences and context.

16. Performing a relevant and accurate examination

17. Performing appropriate special investigations where indicated, based on current evidence and balancing risks, benefits and costs

18. Formulating a bio-psycho-social assessment of the patient's problems, informed, amongst others, by clinical judgment, epidemiological principles and the context

19. Communicating effectively with patients to inform them of the diagnosis or assessment and to seek consensus on a management plan

20. Establishing priorities for management, based on the patient's perspective, medical urgency and context

21. Formulating a cost-effective management plan including follow-up arrangements and re-evaluation

22. Formulating a management plan for patients with family-orientated or other social problems, making appropriate use of family and other social and community supports and resources.

23. Appling technology cost-effectively and in a manner that balances the needs of the individual patient and the greater good of the community.

24. Incorporating disease prevention and health promotion.

25. Effectively managing concurrent, multiple and complex clinical issues, both acute and chronic, often in a context of uncertainty.

26. Demonstrating a patient centred approach to management using collaborative decision making

27. Including the family in management and care of patients whenever appropriate

28. Demonstrates a commitment to building continuity of care and on-going relationships with patients as well as an understanding of the chronic care model

29. Demonstrates the ability to provide preventive care, using primary, secondary, and tertiary prevention as appropriate, and to promote wellness

30. Demonstrates ability to provide holistic palliative & terminal care

31. Recognising and managing discord in relationships impacting on health, using appropriate tools e.g. genograms, ecomaps where necessary to identify potential problems

32. Collaborating and consulting with other health professionals

33. Co-ordinating the care of patients with multiple care providers

34. Demonstrating appropriate record keeping

35. Performing effectively and safely the technical and surgical skills necessary for functioning as a generalist.

Facilitate the health and quality of life of the family & community.

36. Knowing the resources available in the community and being able to co-ordinate and integrate team efforts.

37. Considering the family in assessment and engaging the family in management at an appropriate level

38. Providing family- and community-oriented care to patients

39. Conducting home visits when necessary

40. Demonstrating an understanding of the concept of and an ability to work in a "community"

41. Demonstrating the ability to identify community health problems and make a „community diagnosis'

42. Ensuring co-ordination of care and that the holistic needs of a patient are being addressed at any level of care

Facilitate the learning of others regarding the discipline of family medicine, primary health care, and other health-related matters

Demonstrating the role of the family physician as a teacher, mentor or supervisor by:

43. Describing relevant principles of adult education and learning theory

44. Conducting effective learning conversations in the clinical setting (clinical mentoring)

45. Using educational technology effectively

46. Making an effective educational presentation

Conduct all aspects of health care in an ethical and professional manner

47. Demonstrate an awareness of the legal and ethical responsibilities in the provision of care to individuals and populations by: Identifying and defining an ethical dilemma using ethical concepts

48. Applying a problem solving approach in which the law, ethical principles and theories, medical information, societal and institutional norms and personal value system are reflected

49. Formulating possible solutions to the ethical dilemma

50. Implementing these solutions in order to provide health care in an ethical, compassionate and responsible manner that reflects respect for the human rights of patients and colleagues

#### Learning outcomes not to be assessed in the portfolio (11)

National FM Training Outcomes

1. Working effectively as a member of the district health care team, in any sector

2. Demonstrating the ability to contribute to the management of a facility, sub-district and professional practice.

3. Demonstrating the ability to manage and motivate personnel

4. Demonstrating leadership skills within the context of a team

5. Involving others and planning an integrated approach to addressing problems identified in a community

6. Influencing attitudes in a community towards safer health practices

7. Working together with patients in resolving issues relating to public or private organisations which impact on patients' well-being

8. Speaking on behalf of patients and communities when required

9. Demonstrates professional values in relationship to society: e.g. strives for equity in health care delivery, strives for quality in health care delivery, stands up for human rights of patients and colleagues

10. Demonstrates professional values in interpersonal relationships: e.g. deals courteously with patients, colleagues and the public, having regard for cultural issues and individual dignity

11. Demonstrates professional values in personal behavior: e.g. delivers health care of a consistent high standard irrespective of his/her own perceptions or prejudices, and the background (with respect to gender, ethnicity, religion or sexual orientation) of his/her patient

#### Learning outcomes without consensus-also excluded from the portfolio (24)

National FM Training Outcomes

1. Demonstrating responsible and efficient methods of self-management and self-care

2. Describing and applying the applicable laws with respect to employment practices, labour relations, accounting and running a dispensing practice

3. Interpreting basic financial statements, understanding and applying principles of budgeting, health economics, tax management and principles of financial planning

4. Planning viable health services in a systematic and rational way, incorporating the appropriate use of resources, including human and material resources.

5. Demonstrating an understanding of the principles of the district health system, in the context of the national health system.

6. Facilitating risk management processes

7. Facilitating the development and implementation of a strategic plan

8. Dealing with conflict (with peers, staff and/or patients)

9. Demonstrating sound clinical reasoning at every point in the consultation

10. Counselling patients with regard to a variety of distressing situations such as dreaded diseases and loss, and the need to make difficult decisions.

11. Referring patients to practitioners who are more appropriately qualified than he/she is to manage certain conditions.

12. Demonstrates the ability to make a functional assessment of a patient with impairment or disability and enable their rehabilitation

13. Demonstrates an understanding of the emotional and physical aspects of pregnancy, birth, childhood, adolescence, young adulthood, adulthood and aging

14. Demonstrating an awareness of socio-economic and environmental determinants of ill health and the limits of the biomedical approach to addressing these

15. Demonstrating surveillance skills and an understanding of the processes and procedures for monitoring the health of a community

16. Demonstrating the ability to engage in appropriate community-based research

17. Supporting patients and communities in standing up for their health rights

18. Using research findings to inform health interventions and advocacy

19. Promoting intersectoral interventions that improve the health of a community

20. Assessing the learning needs of others and planning educational activities

21. Facilitating small group learning

22. Eliciting course evaluation and feedback from participants or students

23. Applying the principles of student assessment

24. Applying evidence to the content and methods of teaching

### Assessment methods

Most panel members recommended a number of assessment methods for the various outcomes, with many outcomes possibly to be assessed by more than one method. The panel recommended that the following types of evidence and assessments be included in the portfolio:

• A learning plan and reflection on progress at the end of each rotation or twice per year.

• An evaluation of performance by the supervisor at the end of each rotation or twice per year.

• Written assignments, for example to assess the outcome "Demonstrate an awareness of the legal and ethical responsibilities in the provision of care to individuals and populations by identifying and defining an ethical dilemma using ethical concepts."

• Reports of health service meetings that were personally attended and that dealt with clinical governance, such as mortality and morbidity meetings, patient safety, or monitoring and evaluation meetings.

• Multisource feedback evaluations of the registrar's performance, for example to assess the outcome "Work with people in the health care team to create an optimal working climate by communicating and collaborating effectively with members of the health care team and peers."

• Direct observation, feedback and evaluation by the supervisor, for example to assess the outcome "Evaluate a patient according to the bio-psycho social approach by taking a relevant history in a patient-centred manner, including exploration of the patient's illness experiences and context".

• A logbook of competency to perform clinical skills and procedures.

• Feedback forms from training or educational activities performed by the registrar.

### Principles relating to the portfolio

The panel supported 26 of the 27 principles, but could not reach consensus on whether the portfolio should reflect on the relationship between the supervisor and registrar. The panel felt the portfolio would implicitly provide information on the relationship, but that it did not have to explicitly document this.

#### Principles relating to the use of the portfolio

Portfolio characteristics:

1. A portfolio summary will form part of the CMSA Part 1 examination.

2. The summary is supported by a comprehensive portfolio, not submitted, but regularly updated and formatively engaged with by the registrar and supervisor.

3. It demonstrates reflective learning, going beyond a logbook of activities.

4. It illustrates competency as a family physician to the CMSA and the South African employment market.

5. It is a stimulating, engaging, life-long learning journey, teaching the registrar to become a reflective practitioner.

6. It should change clinical practice, improve care for people in communities, and develop the doctor into a mentor.

7. It must be simple, user-friendly, striving towards less paperwork.

8. The format aims towards an electronic database, with a hard copy back-up.

9. It should eventually be web-based, with the registrar and supervisor having secure access.

10. There should be prompts, with flexibility, e.g. weekly reflections, monthly critical incident reports, supervisor meetings, and 3-monthly learning plans.

Supervisor-registrar relationship:

11. Implicitly linked to the portfolio is the close working and learning relationship between the registrars and their supervisors.

12. Meeting with the registrar every 2 to 4 weeks is a realistic expectation from the supervisor.

13. The registrar is surrounded by a "supervisor team" of peers, family physicians, other specialists, managers, nurses, allied health professionals, patients, and community.

14. While it is implicit that training is ongoing and part of working, 6 hours a week of dedicated time must be set aside for more focussed teaching, research and completion of the portfolio.

15. Honesty between supervisors and registrars is important, including the ability to say that progress is not as expected, and how to improve it.

16. Supervisors should be accredited as competent according to set criteria.

17. The portfolio includes space for the registrar to give feedback on the supervision process.

Assessment issues:

18. Regular recorded meetings with the supervisor are used to set a learning agenda and evaluate progress so that poor competency is detected early.

19. The portfolio contributes significantly towards the CMSA examination mark.

20. Competencies are graded on a Likert-type scale, often with a global score, with recommendations, allowing registrars to improve on low score areas.

21. The portfolio allows for entries by different supervisors, as well as a number of entries by the same supervisor, to increase reliability.

22. The portfolio encourages feedback and reports not only from doctors, but also from nurses, allied health professionals, managers, and patients.

23. An indication of progress should be recorded at the end of each rotation, as well as the end of each year.

24. This progress report is done by the registrar.

25. There should also be an overall report of progress by the supervisor.

26. This report should include a form of Likert scale to grade the overall progress, and qualitative, honest feedback and recommendations for specific areas.

## Discussion

### Key findings

This was the first attempt to develop a learning portfolio at a national level. The importance of the portfolio as a form of assessment is reflected in the finding that 50 (59%) of the national outcomes were assigned to the portfolio as the preferred method of assessment. The panel also affirmed 26 (96%) of the suggested principles which were derived from the WONCA workshop and literature.

The 35 learning outcomes which were left to other ways of assessment, or where no consensus were obtained, probably illustrate that some of the outcomes are very difficult to assess, and may not even be assessed. Many responders had a rather maximalist idea of everything that can (and therefore should) be accessed through the portfolio. Some comments from the experts included, "Important, but difficult to assess.", and "Should be captured and be part of the portfolio, but should not be assessed." Some of these outcomes include:

Working effectively as a member of the district health care team

Demonstrating the ability to contribute to the management of a facility, sub-district and professional practice.

Demonstrating the ability to manage and motivate personnel

Demonstrating leadership skills within the context of a team

Involving others and planning an integrated approach to addressing problems identified in a community

Influencing attitudes in a community towards safer health practices

Dealing with conflict (with peers, staff and/or patients)

Demonstrating sound clinical reasoning at every point in the consultation

Counseling patients with regards to a variety of distressing situations such as dreaded diseases and loss, and the need to make difficult decisions.

Referring patients to practitioners who are more appropriately qualified than he/she is to manage certain conditions.

Demonstrates the ability to make a functional assessment of a patient with impairment or disability and enable their rehabilitation

Demonstrates an understanding of the emotional and physical aspects of pregnancy, birth, childhood, adolescence, young adulthood, adulthood and aging

The assessments of the identified outcomes in the portfolio will form part of the assessments methods used in the examinations of the CMSA and family medicine departments, including OSCEs, MCQs, MEQs, simulated or real consultations, orals, and the research assignment, which will assess many of the outcomes not captured in the portfolio.

A major concern was that the portfolio could become an unwelcome burden on supervisors and registrars. Principle 7 related to this: "It must be simple, user-friendly, striving towards less paperwork." This is well recognised across the world, where the recommendation is clearly to make the portfolio "lean and mean", rather than thick and comprehensive [25,27]. The portfolio should capture a sample of what took place that is sufficient to provide evidence of learning and not record every possible activity [25,27]. It will be important to simplify the portfolio, and not try to assess everything that can be assessed by way of the portfolio.

It was noted that there is already a large amount of formal learning and teaching taking place by way of the various university training programmes. Where these assignments and assessments are relevant to the portfolio, they can simply be summarised or included in the portfolio without the need for duplication. More significantly, perhaps, is the large volume of learning at the bed-side or in the consultation room, which often goes unnoticed in the daily work. It will be a challenge for registrars to notice and maximise the potential for learning in these moments of uncertainty and to embrace conversations that enhance their learning as well as solve their immediate clinical problems [23,24,28]. Another challenge will be to routinely record these learning moments in a manner not unlike journaling, or keeping a diary [24]. It is as we write our thoughts down that we start to understand and believe in what we do. This allows for correction, improvement, and growth [29].

### Strengths and weaknesses of this study

The Delphi method enabled a broadly representative panel from across the country to be included and ensured participation of the key stakeholders in the development of the portfolio. It should be noted however that participation was higher from four of the eight universities.

The revision of the national learning outcomes coincided with round 3 of the Delphi process and had to be accommodated in a 4^th ^round. This could potentially have negatively affected the Delphi process, however the revised national outcomes were broadly similar in content and meaning to the ones tested in Delphi rounds 1-3. It was possible to identify the few truly new concepts and these were easily taken up in round 4, without losing the flow and validity of the Delphi process.

It became clear that many panel members had different understandings of commonly used terms in the questionnaires such as supervisor, mentor, logbook, portfolio, and that this may have influenced their opinion. This was partly addressed through the feedback from panel members, and subsequent to the Delphi process whilst running workshops at the universities on the use of the portfolio, reflective learning, and supervision.

### Implications and recommendations

Following the Delphi process the findings of the study were presented to the eight heads of family medicine in a 1-day workshop. They were also part of the experts who participated in the Delphi rounds. This allowed the heads to make sense of and take ownership of the findings. It also allowed a more practical discussion of how these findings should be taken forward. One major issue was the exact frequency and type of assessment for each item in the portfolio, which were elicited by way of open questions in the Delphi. The frequency of assessment for all the learning outcomes was kept to a minimum, appreciating the huge clinical workload of registrars in South African district hospitals, after hours calls, pressure to complete their research assignments, and allowing time for themselves and their families. The thinking was that the registrar and supervisor should be encouraged to make fewer, but more authentic assessments. The workshop also made a final decision on the type of assessment to be used when more than one option was recommended in the findings.

Mostly they decided that registrars should document evidence of learning for a particular training outcome once or twice during the year, or at the beginning and end of clinical rotations. Registrars could be selective in what was included in the portfolio as evidence of learning.

There were only two instances where they suggested that the frequency of assessment should be higher. Ten direct observations per year of patient interactions (including consultations, procedures, and teaching {patients, colleagues, or community} events) were required as well as a minimum of two hours per month of more formal educational meetings with their supervisor. This makes sense, as it is self-evident that the doctor-patient interaction/consultation is a core element in the discipline of family medicine and that eight to ten assessments increases validity [30]. The educational meetings between the registrar and the supervisor is also a critical part of the registrar's learning [30].

Only one or sometimes two assessment methods (tools) were recommended per item and examples of such tools were included in the portfolio guide. Of course registrars and supervisors are free to use other tools, but again, the aim was simplicity and a degree of national uniformity. For the same reasons there was agreement to have a combination of a global rating and/or a simple Likert scale for most learning events.

Following this workshop a national postgraduate Learning Portfolio (see additional file 2) and Portfolio Guide (see additional file 3) for Family Medicine training in South Africa was distributed to all eight medical schools and the College of Family Physicians [7]. The eight departmental heads and the CMSA have agreed to facilitate the implementation of the national portfolio in their postgraduate training programmes.

Training in the use of the portfolio, with a focus on learning, reflection, and supervision, has been undertaken with registrars and supervisors in a number of universities.

The portfolio will be further refined, and tested with a sample of family medicine registrars from a number of medical schools in South Africa. Qualitative feedback (questionnaires and semi-structured interviews) on the use of the portfolio will be obtained from registrars, family physicians, other supervisors and managers in terms of the tool's educational impact, acceptability, and perceived usefulness for assessment purposes. A final portfolio assessment tool that will satisfy the requirements of the CMSA will then be devised. Each university's training programme would then adapt the national portfolio to dovetail with their local formative and summative assessment needs, whilst retaining the CMSA requirements.

## Conclusion

This was the first attempt to reach consensus on the development of a national portfolio for family medicine training in South Africa. Consensus was reached on 50 items to include, and 26 principles relating to the portfolio. A draft national portfolio and portfolio guide have been developed and distributed to all the medical schools in the country. Further revision and testing with registrars in training is underway, with the aim to deliver a final portfolio in the following year.

## Competing interests

The authors declare no competing interests and no funding was sourced for this research.

## Authors' contributions

LJ designed the study, conducted the Delphi rounds and gathered the data, with imputs and supervision from BM and AD. LJ and BM carried out the analysis of the data. LJ drafted the paper and all authors contributed equally to the discussion and conclusion. All authors read and approved the final paper.

## Pre-publication history

The pre-publication history for this paper can be accessed here:

http://www.biomedcentral.com/1471-2296/13/11/prepub

## Supplementary Material

Additional file 1 **Delphi Round 1 Questionnaire**. Click here for file

Additional file 2 **Portfolio version 1**. Click here for file

Additional file 3 **Portfolio Guide version 1**. Click here for file
