# Supplementary material for: Development of a portfolio of learning for postgraduate family medicine training in South Africa: a Delphi study
Source: BMC Fam Pract. 2012 Mar 3;13:11. doi: 10.1186/1471-2296-13-11 (PMC3317832; doi:10.1186/1471-2296-13-11)
Supplement: Additional file 1 — Delphi Round 1 Questionnaire. [file 1471-2296-13-11-S1.PDF]

Delphi Questionnaire 1

Defining the key contents and assessment criteria for the learning portfolio for family medicine in the South African context

You have been nominated as someone who would be able to give valuable input into the development of a learning portfolio for family medicine training in South Africa. You are invited to participate in this study that will seek to define the key items and best ways to assess these items, as part of a standardised learning portfolio for family medicine in South Africa.

The Colleges of Medicine of South Africa (CMSA), and also the College of Family Physicians in South Africa, with the eight Family Medicine Departments / Divisions, want to standardise the exit examination and assessment of prospective family medicine specialists. The aim is to improve the health of communities through better training and education of family physicians, thus ensuring competence in the context of the district health system in South Africa. The project is part of a PhD, which seeks to establish a standardised, reliable, simple, valid, and acceptable learning portfolio for all family medicine training in South Africa.

A number of issues with regard to which items to include, and in particular how to weigh, or assess these items, have been workshopped at the WONCA African Regional Conference in October 2009. Similarly, a number of University Departments of Family Medicine are already using learning portfolios and have contributed to the questions raised in this project.

The research project will use an electronic Delphi technique to elicit the opinions of a panel of 60 experts between January - April 2010. Consensus will be defined as 70% of the panel reaching agreement on the relevance of each item to the assessment of competency of family medicine registrars as part of a portfolio. Relevance is defined as a portfolio entry that is valuable and useful to determine competence in a family medicine registrar. For each portfolio entry you will be asked to decide if the item is relevant or not for assessment in a learning portfolio format, or whether the item should be modified. Some items may be assessed better by other methods, e.g. OSCEs, orals, or written papers. The questionnaires will also seek to find the best way of assessing the portfolio items, in order to create validity, i.e. how do we all agree on how to “score” the items in the portfolio, in order to predict competence in the workplace.

You are being asked to participate as a member of this expert panel via e-mail. Your opinion will be obtained through your response to a questionnaire and will be collated and analysed anonymously by a research assistant. Following each round the questionnaire will be modified according to your responses as a group and you will be asked to respond again on items where no consensus were reached or where new items have been suggested. Feedback on the previous group response will also be given. We anticipate that you will be asked to respond 3 times and that the size of the questionnaire will decrease with each round.

The members of the panel have been selected according to the following categories of expertise:

- Supervisors responsible for training of family medicine registrars
- Family physicians or general practitioners, including those in academia, who are participating in or managing family medicine training programmes
- Senior family medicine registrars or recently qualified family physicians.

If you are willing to participate, please complete the attached consent form and questionnaire 1, and return it by email, ljenkins@pgwc.gov.za or fax to myself, Dr Louis Jenkins, 0865135729, by 15 March 2010.

If you have further queries regarding the research please contact me, Dr Louis Jenkins, at ljenkins@pgwc.gov.za, or cell 0837951065, or Prof Bob Mash, at rm@sun.ac.za or 021-938-9170.

I thank you very much in anticipation of your time and commitment to this project.

Yours sincerely,

Louis Jenkins

Consent form to participate in the research project:

Defining the key contents and assessment criteria for the learning portfolio for family medicine in the South African context

Researchers: Dr Louis Jenkins, Division of Family Medicine and Primary Care, Stellenbosch University; Prof Bob Mash, Division of Family Medicine and Primary Care, Stellenbosch University; and Prof Anselme Derese, Department of Family Medicine and Primary Healthcare, Centre for Education Research & Development, Faculty of Medicine & Health, Sciences Ghent University, Ghent (Belgium).

I confirm the following:

1. I am invited to participate in this research project, which is being endorsed by the CMSA.

2. Having read the letter of invitation the following aspects are understood by me:

- Aim of the project
- I will be required to respond electronically to 3 questionnaires
- My replies will be confidential and analysed anonymously
- The research findings will be disseminated to the panel
- I am participating of my own free will
- I will receive no remuneration for my contribution
- Any queries regarding the project have been addressed by the researchers
- The project has been approved by the Committee for Human Research at Stellenbosch University and will be conducted according to the ethical guidelines and principles of the international Declaration of Helsinki, South African Guidelines for Good Clinical Practice and the Medical Research Council Ethical Guidelines for Research.

I hereby consent voluntarily to participate in the above mentioned research project

Name ……………………………………………………………………. Signed at ………………………………………………….. on ……………………..……….2010

……………………………………………………………….

Signature

Email ……………………………………………………. Cell ………………………………………………….

Tel ………………………………………………………. Fax …………………………………………………

Please return by fax to Louis Jenkins 0865135729, or e-mail ljenkins@pgwc.gov.za

Contact details:

Prof Bob Mash

Dept. of Family Meds and Prim Care

Stellenbosch University

Box 19063

Tygerberg

7505

Tel: 27 21 938 9170

Fax: 27 21 938 9153

Email: rm@sun.ac.za

Dr Louis Jenkins

Department of Family Medicine

George Complex

P/Bag X6530

George, 6534

Southern Cape

Tel: 0837951065

Fax: 0865135729

Email: ljenkins@pgwc.gov.za

Prof Anseleme Derese

Department of Family Medicine and Primary Healthcare
Centre for Education Research & Development, Faculty of Medicine & Health Sciences Ghent University
University Hospital 3 K3
De Pintelaan 185
B-9000 Ghent (Belgium)
Tel +32 9 332 5571
Fax +32 9 332 4990
Mobile +32 475 781402
E-mail: anselme.derese@ugent.be

Blueprinting of the Learning Portfolio for registrars in Family Medicine in South Africa (according to the CMSA learning outcomes)

Items that must be included in the learning portfolio (61):

All these items are taken from the agreed national outcomes for family medicine training and have been identified as potentially relevant to portfolio assessment.

Please rate each item in the list as:

A: "must be included for assessment in the portfolio", or
B: "would be good to include, but not sure how to assess", or
C: "should be left out ~ can be assessed better in another way".

Please write down your answer to each of the following questions:

- How to best assess the specific item? (e.g. direct observation, video/audio recording, peer review, written assignment, group educational meeting, interaction with supervisor, written self-reflection) and
- How to record the assessment? (e.g. Likert scale (i.e. 0-5), grade (i.e. out of 100%), global rating (i.e. adequate/not adequate)).

Spaces are also given for your additional comments or items.

Outcome	Rating of item: A,B or C	How to assess?	How to record the assessment?	Other comments?
**1. Manage him/herself and his/her practice (in public or private sector) effectively (9).**				
Demonstrate responsible and efficient methods of self-organisation, -management and – care.				
Apply criteria for effectiveness in practice ~ Quality assurance cycle<br>Plan and conduct a practice audit				
Be able to participate in the management of a professional practice.				
Work effectively as a member of the primary and / or secondary health team (public and private sector)				
Communicate effectively with members of the health care team (public and private sector)				
Demonstrate the ability to manage and motivate personnel				
Implement a strategic plan				
Demonstrate leadership skills within the context of a team				
Deal with conflict (with peers, staff and / or patients) successfully				

Outcome	Rating of item: A,B or C	How to assess?	How to record the assessment?	Other comments?
**2. Evaluate and manage patients with both undifferentiated and more specific problems cost-effectively according to the bio-psycho-social approach (15).**				
Take a relevant history in a patient-centred manner.				
Perform a relevant and accurate examination and appropriate special investigations.				
Formulate a bio-psycho-social assessment of the patients problems				
Communicate effectively with patients to inform them of interpretation of findings and to seek consensus on a management plan.				
Formulate a cost-effective management plan including follow-up arrangements, appropriate referral and re-evaluation.				
Formulate a management plan for patients with family-orientated or other social problems.				
Apply science and technology efficiently and in a manner demonstrating responsibility towards the health of the patient and the demands of society.				

Outcome	Rating of item: A,B or C	How to assess?	How to record the assessment?	Other comments?
Perform technical and surgical skills necessary for functioning as a generalist: The portfolio should include a logbook which records the actual number of procedures done, or				
Perform technical and surgical skills necessary for functioning as a generalist: The portfolio should include global ratings of competence (adequacy) in the various skills, or				
Perform technical and surgical skills necessary for functioning as a generalist: The portfolio should include a combination of the above 2 outcomes.				
Perform technical and surgical skills necessary for functioning as a generalist: The portfolio should include summaries/reflections of M&M (mortality and morbidity) meetings.				
Demonstrate a patient centred approach to management				
Include the family in management.				
Recognise and manage discord in relationships impacting on health, using appropriate tools e.g. genograms, ecomaps where necessary to identify potential problems.				

Outcome	Rating of item: A,B or C	How to assess?	How to record the assessment?	Other comments?
Demonstrate an awareness of the process of the consultation.				
**3. Facilitate the health and quality of life of the community (9).**				
Know the resources available in the community and be able to co-ordinate and integrate team efforts.				
Understand the roles of different team members				
Apply principles of cost-effectiveness in the utilisation of resources.				
Be able to define and work with a "community"				
Demonstrate an awareness of socio-economic and environmental causes of ill health and the limits of the biomedical approach to addressing these.				
Surveillance skills: know the processes and procedures for monitoring community health.				
Involve others and plan an integrated approach to				

Outcome	Rating of item: A,B or C	How to assess?	How to record the assessment?	Other comments?
addressing problems identified in the community.				
Communicate observations of local conditions to relevant parties.				
Influence attitudes of the community towards safer health practices.				
**4. Recognise, evaluate and reflect on personal and professional strengths and weaknesses in order to appropriately change professional practice and behaviour (8).**				
The portfolio should capture previous relevant experience, prior to joining programme.				
Perform an audit of personal professional practice by, for instance peer review, to:				
Identify and assess own learning needs and objectives according to the 6 CMSA exit level outcome categories, and weighing/prioritizing registrar's own outcomes/needs envisioned within these categories.				
Formulate plan to address learning needs.				

Outcome	Rating of item: A,B or C	How to assess?	How to record the assessment?	Other comments?
Implement above plan, mobilising resources and help where appropriate.				
Evaluate success of actions: Reflections on how well outcomes/objectives have been achieved/not achieved.				
Critically review research articles.				
Demonstrate the implementation of research and literature review findings in the management of problems in practice. by, for instance developing protocols for the practice.				
**5. Educate, consult and advise health care professionals, health care workers and institutions on the discipline of family medicine and on health related issues (4).**				
Demonstrate teaching skills under the supervision of academic staff of a university department.				
Demonstrate integration of knowledge and skills of teaching in a clinical setting.				

Outcome	Rating of item: A,B or C	How to assess?	How to record the assessment?	Other comments?
Demonstrate the ability to give clear explanations on conditions and interventions to enable informed mutual decision making with patients.				
Advocating for patients in resolving issues relating to public or private organisations which impact on the patients' well being.				
**6. Conduct all aspects of health care in an ethical, compassionate and responsible manner (6).**				
Refer patients to more appropriately qualified practitioners in the case where he/she is not able to manage a patient optimally.				
Demonstrate an ability to identify ethical dilemmas form everyday professional practice, formulate a framework to assess the problem and arrive at a balanced outcome or solution.				
Demonstrate an awareness of his/her own value system.				

Outcome	Rating of item: A,B or C	How to assess?	How to record the assessment?	Other comments?
Delivers health care of a consistent high standard irrespective of his/ her own perceptions or prejudices, and the background (with respect to gender, ethnicity, religion or sexual orientation) of his/her patient.				
Deals courteously with patients, colleagues and the public, having regard for cultural sensitivities and individual dignity, displaying unconditional positive regard towards patients ~ cultural competency.				
Strives for equality in the distribution of resources for health care delivery.				
**ADDITIONAL ITEMS (10)**				
Personal Information				
Record of attachments/rotations as registrar				
Discipline-specific certificates and diplomas				
Congresses, seminars and workshops attended				
List of presentations and publications				
Critical incidents reports (e.g. M&Ms)				
Extra-curricular professional activities				
Personal Reading				
Use of the internet				

Outcome	Rating of item: A,B or C	How to assess?	How to record the assessment?	Other comments?
Declaration of authenticity of portfolio completion.				

Principles and Processes (27):

Please comment on whether you agree or disagree with the following principles and processes pertaining to the learning portfolio. If you disagree, please give your comment or opinion on how you would view this principle. Please also add any other principles and processes that should be included, in the open rows provided.

Portfolio characteristics:	Agree	Disagree	Comment/Opinion if disagree
1. In order to enter the CMSA Part 1 examination (after 3 years of training), a portfolio summary will be submitted, which will form part of a summative assessment process.			
2. This portfolio summary will be supported by a more comprehensive and extensive portfolio, not necessarily submitted, but meticulously updated annually by the registrar and regularly engaged with by their supervisors in a formative assessment process.			

3. The portfolio is not merely a logbook of work done / activities. It must also demonstrate insight and reflective learning.			
4. It should illustrate competency as a family physician, to the point that the CMSA and any prospective employer would be confident to accept and employ the doctor as a specialist family physician in the SA context.			
5. The process should be stimulating and engaging, part of a life-long learning journey, teaching the registrar to become a reflective practitioner.			
6. It should change clinical practice/behaviour, improve care for people in communities, and develop the doctor into a mentor him/herself (as opposed to a paper exercise to satisfy assessment requirements).			
7. It must be simple, user-friendly, and strive towards less paperwork rather than more.			
8. The format should aim towards an electronic database. The registrar and supervisor can access and enter data. The registrar must keep a hard copy back-up.			
9. The format should eventually be web-based,			

with the registrar and supervisor having secure access.			
10. There should be prompts, e.g. weekly reflections, monthly critical incident reports, regular supervisor meetings, 3-monthly learning plans.			
**Supervisor (mentor) ~ registrar commitments (relationship):**	**Agree**	**Disagree**	**Comment/Opinion if disagree**
11. Implicitly linked to the portfolio is the close working and learning relationship between the registrars and their mentor/supervisor.			
12. Meeting with the registrar every 2 to 4 weeks is a realistic expectation from the mentor/supervisor.			
13. The registrar is surrounded by a "supervisor team" of peers, family physicians, other specialists, managers, nurses, allied health professionals, patients, and community.			
14. While it is implicit that training is ongoing and part of working, 6 hours a week of dedicated			

<u>time</u> must be set aside for more focussed teaching, research and completion of the portfolio.			
15. <u>Honesty</u> between supervisors/mentors and registrars is important, which includes having the ability to say that progress is not as expected, and how to improve it.			
16. The portfolio should reflect the <u>relationship</u> between the mentor and the registrar.			
17. Supervisors and mentors should be <u>selected and accredited</u> as competent according to set criteria.			
18. The portfolio should include space for the <u>registrar to give feedback</u> on the supervision process.			
**Assessment issues:**	**Agree**	**Disagree**	**Comment/Opinion if disagree**
19. Regular <u>meetings with the mentor</u> should be used to set a learning agenda and evaluate progress so that poor competency is detected			

quickly and early on. These meetings are recorded in the portfolio.			
20. The portfolio should contribute significantly towards the CMSA examination mark.			
21. Competencies should be graded on a Likert-type scale (1 to 10), e.g. as:<br>Knows theoretically (1-2), Has observed this skill (3-4), Has performed the skill several times (5-6), Can perform the skill independently (7-8), Can teach the skill (9-10), or<br>Not adequate (needs more work) ~ Adequate (but could do better) ~ Excellent (cannot do much better)?			
22. The portfolio should allow for multiple supervisors' entries (different supervisors, as well as a number of entries by the same supervisor) ~ will increase reliability.			
23. The portfolio should encourage feedback and reports not only from doctors, but also from nurses, allied health professionals, managers, and patients.			
24. An indication of progress should be recorded at the end of each rotation, as well as the end of each year.			

25. This progress report should be done by the registrar.			
26. There should also be an overall report of progress by the mentor/supervisor.			
27. This report should include a form of Likert scale to grade the overall progress, e.g. Not sufficient, Slow progress, Quite acceptable, Very good, Excellent.			

Thank you very much for your time and input. You will be notified about the results of questionnaire 1 very shortly.
