# Supplementary material for: Development of a portfolio of learning for postgraduate family medicine training in South Africa: a Delphi study
Source: BMC Fam Pract. 2012 Mar 3;13:11. doi: 10.1186/1471-2296-13-11 (PMC3317832; doi:10.1186/1471-2296-13-11)
Supplement: Additional file 2 — Portfolio version 1. [file 1471-2296-13-11-S2.PDF]

CMSA

PORTFOLIO OF LEARNING

Fellowship

of the

College of Family Physicians of South Africa

FCFP(SA)

AND

Master of Medicine in Family Medicine

MMed (Fam Med)

PORTFOLIO OF LEARNING

CONTENTS

SECTION 1 Personal Details

SECTION 2 Purpose of Portfolio

SECTION 3 Learning Outcomes

SECTION 4 Learning plans

SECTION 5 Rotations

SECTION 6 Educational Meetings

SECTION 7 Observations

SECTION 8 Assignments

SECTION 9 Logbook

SECTION 10 Addendum / Lists

SECTION 11 End of Year Assessment

SECTION 12 Declaration of Completion of Portfolio

SECTION 1

PERSONAL DETAILS

SURNAME:..

FIRST NAMES:..

ID NUMBER:..

HPCSA NUMBER: ....

TRAINEE POST NUMBER: ....

NAME OF TRAINING COMPLEX: ....

NAME OF COMPLEX TRAINING
COORDINATOR:... ………………………………

PREFERRED POSTAL ADDRESS: ....

……………………………………………………………………………………………

……………………………………………………………………………………………

EMAIL ADDRESS:..

TELEPHONE NUMBER: (Work):……………………………….(Home): ………………………

CELLPHONE NUMBER:..

FAX NUMBER: ....

UNDERGRADUATE MEDICAL QUALIFICATIONS

UNIVERSITY: ....YEAR:………………………

INTERNSHIP

HOSPITAL: .... YEARS:………………………

TRAINING EXPERIENCE:...

....

....

OTHER REGISTERABLE POST-GRADUATE QUALIFICATIONS

DIPLOMA/DEGREE:.. .....YEAR:………………………

INSTITUTION: ....

DIPLOMA/DEGREE:.. .....YEAR:………………………

INSTITUTION: ....

CHRONOLOGICAL POST-INTERNSHIP PROFESSIONAL EXPERIENCE
(Prior to commencement of Family Medicine Registrar post)

POST	HOSPITAL/PRACTICE	DEPARTMENT	COUNTRY	DATES

SECTION 2

PURPOSE OF THE PORTFOLIO

What is the Portfolio?

Your portfolio provides evidence of learning in the workplace during your time as a registrar in family medicine. It allows you to demonstrate that you have met the outcomes of the training programme. Many of these outcomes are best assessed in the portfolio.

Guide to the Portfolio

You and your supervisor should have been provided with a guide to creating your portfolio, which will assist both yourself and your supervisor with its development. If you do not have the guide please ask your supervisor to provide it and read through the guide yourself.

The learning portfolio for Family Medicine training in South Africa has been developed through an extensive process of consultation and consensus between all eight Family Medicine academic departments in the country. In terms of national training outcomes for Family Medicine, 5 unit standards have been agreed upon. Within these 5 unit standards there are 85 more specific training outcomes. The portfolio does not intend to reflect training and learning in all of these, as some outcomes will be assessed through other means. The 50 outcomes that must be reflected in the portfolio are summarised in a grid below and should be constantly referred to and kept in mind as you work and learn in daily practice.

Purpose of the portfolio

1. To stimulate you to think consciously and objectively about your own training. This is known as *reflective learning*, and is its primary purpose.
2. To document the scope and depth of your training experiences.
3. To provide a record of your progress and personal development as training proceeds.
4. To provide an objective basis for discussion with your supervisors about work performance, objectives, and immediate and future educational needs.
5. To provide documented evidence for the CMSA of the quality and intensity of the training that you have undergone, as a requirement to sit the Part I exam for the FCFP.

The portfolio is not just a logbook of signed procedures undertaken or witnessed. It should contain your written reflections and systematic documentation of your learning experience. It includes opportunities for you to reflect, to explore, to form opinions, and

to identify your own strengths and weaknesses. It allows you to follow your own progress; not only with regard to the training programme, but also in terms of learning goals you have set for yourself. In this way the portfolio provides an opportunity to record and document the subjective aspects of training.

Objectives

The objectives of your portfolio are to:

- develop a structured learning plan
- identify goals and actions required to achieve them
- record progress in achieving those goals
- document personal strengths
- identify areas needing improvement

Who looks at the Portfolio of Learning?

1. **Registrars**. You should interact regularly with your portfolio to ensure it documents your learning on a continuous basis and stimulates you to reflect on your experiences.
2. **Supervisors**. You should meet on a regular basis with your supervisor to develop and reflect on your learning plans, to observe and reflect on your clinical practice and to have a variety of educational meetings. All these activities should be documented in your portfolio. Your supervisor should also review progress with the portfolio during intermittent evaluations of your progress. In this way the portfolio allows a structuring of the supervision process.
3. **CMSA**. The CMSA requires evidence that learning has taken place as part of a structured programme, in order to sit Part I of the FCFP exam. The portfolio is an important piece of evidence for this.

This portfolio is a cumulative record of your personal learning, goals, needs, strategies and activities throughout your training programme. The sections in the portfolio are not exhaustive, but rather an indication of the minimum that you should be doing. You will learn a great deal more than what is written on these pages.

The portfolio does not aim to assess or capture all the competencies needed to be a family physician, nor is it the only way of assessing you. Some competencies or skills will also be tested or validated via other means, e.g. orals, OSCEs, Multiple Choice Questions, assignments and written papers in formal exams.

The portfolio should not become a big additional burden on you and the supervisor. In many instances you can include reports from meetings that you attend as part of your work (e.g. M&M meetings) or assignments that you have done as part of the academic programme for the university(e.g. reflective .writing, assignments, patient studies, clinical audits and community projects). These should not be duplicated as a paper exercise, but should simply be incorporated into the portfolio.

The emphasis is on the *process* of completing the portfolio (in a way that encourages *reflection*), and "the learning journey" rather than "something else that must be done and handed in for marks." Be creative, for example you can include photos of a community project, or letters written as the patient advocate, etc.

Portfolio Completion Criteria

The Portfolio should always be used in conjunction with the ***Regulations and Syllabus for admission to the Fellowship of the College of Family Physicians of South Africa FCFP(SA)***, as may be amended from time to time. See http://www.collegemedsa.ac.za/Documents%5Cdoc_191.pdf (17 pages)

- Entries must at all times be **legible** and, where indicated, supported by the required **signatories** (Supervisors and Heads of Departments and their contact details). Add pages to each Section as necessary. Ensure that your name appears on every page. It is **strongly advised** that you keep an electronic backup copy of all entries, as well as a printed copy, in case of computer failure or theft.
- Each rotation will need to be verified by the relevant Head of Department or Supervisor, including the relevant sections in your logbook (procedures and clinical skills done).
- You must submit your completed portfolio at the end of every year during years 1-3 of your training programme to the head of department, for assessment purposes. In you^{r} 4th year of training, you should have a comprehensive portfolio, with cumulative evidence of learning that has been assessed every year by the university department, and will be part of the admission requirements for the CMSA exams.
- The final portfolio must reach your university head of department ***at least 3 (three) months*** prior to the commencement of the FCFP(SA) Part I Examination, in order for the head to submit a report, which will be sent to the Academic Registrar of the CMSA. Failure to submit the portfolio on time will result in the candidate not being invited to the examination.
- The **Declaration** (Section 12) must be signed by the registrar before submitting the final portfolio at the end of 3 completed years of training.

SECTION 3

NATIONAL UNIT STANDARDS and EXPECTED LEARNING OUTCOMES TO BE ASSESSED IN THE PORTFOLIO

It is important to keep the national training outcomes for Family Medicine in mind while you develop your portfolio. The 5 national Family Medicine Training Unit Standards are broken down into a number of outcomes, of which 50 will be reflected on and assessed in the portfolio. These should help you to develop your personal learning plans.

To remind you and your supervisor to plan appropriately, it is suggested that you mark off what you have completed in the portfolio in the column on "frequency of assessment". This will ensure inclusion of all the outcomes in the portfolio over time.

OUTCOMES TO BE ASSESSED IN PORTFOLIO (50)	Recommended assessment methods	Suggested frequency of assessment
**UNIT STANDARD 1**<br>Effectively manage him/herself, his/her team and his/her practice, in any sector, with visionary leadership and self-awareness, in order to ensure the provision of high-quality, evidence-based care.		
**Manage him/herself optimally by:**<br>1. Addressing his/ her personal learning needs continually by assessing needs and participating in an appropriate programme of learning.	Learning Plan, signed by supervisor	2X/year
2. Demonstrating growth and learning in response to identified needs		
3. Demonstrating willingness to seek help when necessary		
4. Describing activities to enhance self-growth and development		
5. Demonstrating ability to develop his/her own capacity		
**Manage resources and processes effectively by:**<br>1. Planning, implementing and maintaining information- and record-keeping systems.	Continuous assessment form	End of rotations
**Describe, evaluate and manage health care systems by:**<br>1. Demonstrating the ability to plan and conduct a practice audit	Written assignment	Once during programme
2. Implementing ongoing quality improvement activities		
**Facilitate clinical governance by:**<br>1. Critically reviewing research articles and applying the evidence in practice	Written assignment	Once during programme
2. Demonstrating the implementation of research and literature review findings in the management of problems in practice by, for instance, developing protocols for the practice		
3. Adapting and implementing appropriate local, national and international clinical guidelines		
4. Engaging in monitoring and evaluation to ensure high quality care	Report/minutes of M&E meeting	Yearly

5. Implementing rational prescribing and diagnostic testing	Continuous assessment form	End of rotations
**Work with people in the health care team to create an optimal working climate by.**<br>1. Communicating and collaborating effectively with members of the health care team and peers	Multi-source feedback, or Observation by supervisor.	Yearly
**UNIT STANDARD 2**<br>Evaluate and manage patients with both undifferentiated and more specific problems cost-effectively according to the bio-psycho-social approach		
**Evaluate a patient according to the bio-psycho social approach by:**<br>1. Taking a relevant history in a patient-centred manner, including exploration of the patient's illness experiences and context.	Observation by supervisor.<br>(Additionally, a written assignment can be added)	10 Observations / year
2. Performing a relevant and accurate examination		
3. Performing appropriate special investigations where indicated, based on current evidence and balancing risks, benefits and costs		
4. Formulating a bio-psycho-social assessment of the patient's problems, informed, amongst others, by clinical judgment, epidemiological principles and the context		
**Formulate and execute, in consultation with the patient, a mutually acceptable, cost-effective management plan, evaluating and adjusting elements of the plan as necessary by:**<br>1. Communicating effectively with patients to inform them of the diagnosis or assessment and to seek consensus on a management plan		
2. Establishing priorities for management, based on the patient's perspective, medical urgency and context		
3. Formulating a cost-effective management plan including follow-up arrangements and re-evaluation		
4. Formulating a management plan for patients with family-orientated or other social problems, making appropriate use of family and other social and community supports and resources.		
5. Appling technology cost -effectively and in a manner that balances the needs of the individual patient and the greater good of the community.		
6. Incorporating disease prevention and health promotion.		
7. Effectively managing concurrent, multiple and complex clinical issues, both acute and chronic, often in a context of uncertainty.		
8. Demonstrating a patient centred approach to management using collaborative decision making		
9. Including the family in management and care of patients whenever appropriate		
10. Demonstrates a commitment to building continuity of care and on-going relationships with patients as well as an understanding of the chronic care model		
11. Demonstrates the ability to provide preventive care, using primary, secondary, and tertiary prevention as appropriate, and to promote wellness		
12. Demonstrates the ability to provide holistic palliative and terminal care		
13. Recognising and managing discord in relationships impacting on health, using appropriate tools e.g. genograms, ecomaps where necessary to identify potential problems	Written assignment.	Once during programme
14. Collaborating and consulting with other health professionals as appropriate	Continuous	End of rotations

15. Co-ordinating the care of patients with multiple care providers	assessment form.	
16. Demonstrating appropriate record keeping		
17. Performing effectively and safely the technical and surgical skills necessary for functioning as a generalist.	Logbook	Beginning and end of each rotation
UNIT STANDARD 3<br>Facilitate the health and quality of life of the family and community.		
**Integrate and co-ordinate the preventive, promotive, curative, rehabilitative and palliative care of the <u>individual</u> in the context of the family and the community by:**<br>1. Knowing the resources available in the community and being able to co-ordinate and integrate team efforts.	Written assignment	Once during programme
2. Considering the family in assessment and engaging the family in management at an appropriate level		
3. Providing family- and community-oriented care to patients		
4. Conducting home visits when necessary		
**Identify and address problems influencing the health and quality of life of the <u>community</u> in which the family physician works, by:**<br>1. Demonstrating an understanding of the concept of and an ability to work in a "community"		
2. Demonstrating the ability to identify community health problems and make a 'community diagnosis'		
**Be an advocate for individuals and communities to ensure informed decision making on health matters based on evidence by:**<br>1. Ensuring co-ordination of care and that the holistic needs of a patient are being addressed at any level of care		
UNIT STANDARD 4<br>Facilitate the learning of others regarding the discipline of family medicine, primary health care, and other health-related matters		
**Demonstrate the role of the family physician as a teacher, mentor or supervisor by:**<br>1. Describing relevant principles of adult education and learning theory	Feedback from people who were taught, or<br>Observation by supervisor, or<br>Written assignment.	Yearly
2. Conducting effective learning conversations in the clinical setting (clinical mentoring)		
3. Using educational technology effectively		
4. Making an effective educational presentation		
UNIT STANDARD 5<br>Conduct all aspects of health care in an ethical and professional manner		
**Demonstrate an awareness of the legal and ethical responsibilities in the provision of care to individuals and populations by:**<br>1. Identifying and defining an ethical dilemma using ethical concepts	Written ethics assignment	Once during programme
2. Applying a problem solving approach in which the law, ethical principles and theories, medical information, societal and institutional norms and personal value system are reflected		
3. Formulating possible solutions to the ethical dilemma		
4. Implementing these solutions in order to provide health care in an ethical, compassionate and responsible manner that reflects respect for the human rights of patients and colleagues		

SECTION 4

LEARNING PLAN

The meetings with your supervisor to develop and reflect on your Learning Plans need to be documented at least 6-monthly, or at the beginning and end of every rotation. This section must be completed together with the next section (Reflections on rotations), and with your Logbook at hand. See the section in the guide on how to develop your learning plan. You should document your learning plan below and ensure your supervisor has assessed and signed it.
(Remember to make copies of the next 2 pages for new learning plans.)

Period: from …………………………..………. to ………..…………….………

Clinical Rotation: ……………………………………………………………………

A. Learning Objectives:

Relevant prior learning for this clinical rotation:
....

....

……………………………………………………………………………………………

……………………………………………………………………………………………

Learning needs/objectives:
....

....

……………………………………………………………………………………………

……………………………………………………………………………………………

Planned activities to meet these objectives:
....

....

……………………………………………………………………………………………

……………………………………………………………………………………………

Timelines, Support and Resources required to meet these objectives:
....

....

……………………………………………………………………………………………

……………………………………………………………………………………………

Evaluation (how will you know if these objectives have been met, suggested tools):
....

....

……………………………………………………………………………………………

……………………………………………………………………………………………

Registrar:_____________________. Signature:________________ Date:______

B. Supervisor Comments

....

....

....

....

C. Supervisor Assessment (ringed)

Excellent Satisfactory Poor Unacceptable

D. Date of next meeting to review progress / rotation

....

Supervisor……………………………Signature………………………………Date………

SECTION 5

REFLECTION ON ROTATION

(Please make copies and add to your portfolio for every new rotation)

Name of rotation: ____

Rotation starting _____________________ and ending__________________

Name of health facility: ____

Type of health facility (please circle):
PHC District hospital Regional hospital L3 Hospital Other (e.g. TB / Psychiatry)

Clinical area(s) covered in this rotation (please tick all that apply):

Adult medicine	☐	Infectious Diseases (HIV/TB)	☐
Obs & Gynae	☐	Surgery	☐
Paediatrics	☐	Orthopaedics	☐
Anaesthetics	☐	Emergencies	☐
ENT	☐	Eyes	☐
Dermatology	☐	Psychiatry	☐
Other (specify)	☐		

Provide a brief **description** of your duties, patient profile and patient numbers personally managed in this rotation.

Reflect on your **experience** as a registrar working in this facility/department during this rotation, what worked well and what could be improved?

Reflect on your **learning** during this rotation. What has been learnt? What remains to be learnt? (Refer to the Learning Objectives in your Learning Plan.)

Registrar ________________________________<br>(Signature)<br><br>Supervisor:________________________________<br>(Print name) (Signature)<br>Date: ____________	Leave days:

CONTINUOUS ASSESSMENT BY SUPERVISOR

(To be completed by supervisor and discussed with registrar)

Marking scale: 9–10 = excellent; 7–8 = above average; 5–6 = average/satisfactory; 3-4 = below average/unsatisfactory; 1–2 = very weak; N/A = not applicable or don't know

	Score 1 – 10
**KNOWLEDGE**	
• Clinical medicine	
**SKILLS**	
• Clinical record-keeping: case-notes, letters, summaries	
• Rational prescribing and use of medication	
• Rational use of diagnostic tests and resources	
• Co-ordination of patient care with multiple providers	
**PROFESSIONAL VALUES AND ATTITUDES**	
• Approach to ethical and medico-legal issues	
• Punctuality, time keeping and reliability	
• Relationship with other team members	
• Leadership abilities	
• Collaboration or consulting with other health professionals	
**OVERALL ASSESSMENT**	
• Global rating	

Comments from supervisor:

Supervisor’s name: ________________Signature:_____________Date:_____________

SECTION 6

RECORD OF EDUCATIONAL MEETINGS WITH SUPERVISOR

The portfolio at the end of each year should demonstrate engagement with all of the activities below and a minimum of 2-hours formal tuition per month / 24-hours for the year. However, the aim should be to show engagement above the minimum standard.

Use the letters below to record the general focus of the meeting and then describe what was done. The meeting could focus on one of the following learning conversations:

A: Setting a learning agenda (at the beginning and end of a rotation or every 6-months): Reflection on the registrars experience to date, expectations or progress and planning of learning activities and goals for the next period.

B: Intermittent evaluation: For the registrar and trainer to check progress, review the portfolio, discuss any difficulties in their relationship or the organization that impede learning or service delivery, make new plans. Feedback can also be given and received on the programme or registrars performance.

C: Clinical / communication skills: Observation/audio/video-review of communication, consultation or procedural skills and feedback with role-play or simulation. Other clinical skills might also be demonstrated.

D: Case discussions: Reflect on your actual patients through the use of record review, presentation of problem patients or clinical tutorials on specific topics. Reflect on difficult consultations, emotions or ethical dilemmas that arise from your clinical practice or setting.

E: Evidence based practice: Reflect on and critically appraise current journals and original research.

F: Other: For example co-ordination of on-line learning tasks with the on-site professional experience and service priorities i.e. topic for the quality improvement cycle

Please also refer to the section in the guide on educational meetings.

Date	Group or individual meeting	Code letter from list of learning opportunities	Duration (hrs)	Description of content covered / activities / topics	Signature of supervisor
1/1/2011	Group	A	1	Learning plan for rotation in anaesthetics	Example

Date	Group or individual meeting	Code letter from list of learning opportunities	Duration (hrs)	Description of content covered / activities / topics	Signature of supervisor

SECTION 7

OBSERVATIONS OF THE REGISTRAR

This section must include **at least ten (10) observations** of the registrar, during the course of each year. These must include observations of consultations, procedures done, and teaching activities.

A number of Assessment Methods and Tools are available to help with direct or indirect observation. Please see the Portfolio Guide for more information and examples.

SECTION 8

ASSIGNMENTS

Written assignments may be used to provide evidence of learning in any of the following areas (see also the table on outcomes and assessment methods in section 3). Please include any of the following assignments together with their assessment in your portfolio. By the end of the 4 years you should have assignments in all of the following categories. These assignments are usually integrated into the requirements of your academic programme and can just be copied and included in your portfolio:

1. Clinical competence (e.g. patient studies that demonstrate diagnostic reasoning, bio-psycho-social approach)
2. Family-orientated Primary Care
3. Ethical reasoning and medico-legal issues
4. Community-orientated Primary Care
5. Clinical governance
    a. Evidence-based Medicine (e.g.critical appraisal of a journal article, searching for evidence, use of guidelines)
    b. Quality improvement cycle / audit
    c. Significant event analysis (SEA)
    d. Morbidity and mortality meeting reports
    e. Monitoring and evaluation meeting reports

SECTION 9

Logbook

The following tables list the clinical skills that should be acquired or consolidated during the 4-year registrar training in Family Medicine. The list is intended to guide you and your supervisor on what core practical experience and skills training to focus on. **The supervisor** should evaluate your competency **at the beginning and end of the rotation or at least every 6-months (i.e. February and August)**.

It is assumed that while learning these specific skills you will also be exposed to an appropriate spectrum of patients and will be supervised in the relevant clinical assessment, decision making and management.

The skills should be **rated** according to the following definitions from A to D. The rating should be entered in the tables below. If you have not been exposed to a particular clinical area at all during the year or rotation then leave the column blank.

You should also give an **indication of the numbers of a certain procedure done (< 5, 5-10, or >10)**

A: Only Theory:
Only theoretical knowledge regarding the skill's principles, indications, contraindications, performance and complications.
B: Seen or have had demonstrated:
Have theoretical knowledge regarding the skill and have seen or observed the skill demonstrated by someone else
C: Apply/Perform:
Have theoretical knowledge regarding the skill and have performed the skill in question under supervision, at least several times.
D: Routine/Independent:
Have the theoretical knowledge regarding the skill and are competent to perform the skill independently.

Adult medicine

Clinical topic	Clinical skills – aim is D for unshaded skills and C for shaded skills	Numbers done (<5, 5-10, >10)	Grade the registrar 1st assess,2nd assess	
**Adult health - general**	Femoral vein puncture			
	Lumbar puncture			
	Arterial sampling radial artery			
	Blood culture technique			
	Injections - intra-dermal, subcutaneous, intra-muscular, deep intramuscular, sub-conjunctival,			
**Adults-Abdomen**	Interpret the AXR in an adult			
	Proctoscopy			
**Adults-Chest**	ECG - set-up, record and interpret 12 lead ECG			
	Interpret CXR			
	Pleural tap			
	Measure PEF			
	Nebulise a patient			
	Use inhalers and spacers			
	Exercise stress test			
	Perform and interpret office spirometry			
	Pleural biopsy			

Obstetrics and Gynaecology

**Clinical topic**	**Clinical skills – aim is D for unshaded skills and C for shaded skills**	**Numbers done (<5, 5-10, >10)**	**Grade the registrar 1st assess,2nd assess**	
**Antenatal care**	Antenatal growth chart			
	Assess foetal movement / wellbeing			
	Clinical pelvimetry			
	Obstetric ultrasound			
	Amniocentesis			
**Intra-partum care**	Examine progress during labour and use partogram			
	Apply and interpret CTG			
	Assess foetal wellbeing during labour			
	Normal vaginal delivery			
	Assisted vaginal delivery / vacuum extraction / forceps			
	Caesarean section (including ability to do sub-total hysterectomy)			
	Episiotomy and suturing			
	Repair of 3rd degree tear			
	Evacuation of uterus			
	Manual removal of placenta			
	External cephalic version			
**Newborn / Post-partum care**	Resuscitate a newborn			
	Umbilical vein catheterization			

	Assess gestational age at birth			
	Kangaroo mother care			
	Phototherapy			
	Well newborn check			
**Women's health**	Microscopy of vaginal discharge (wet mount, KOH)			
	Endometrial biopsy/sampling			
	Dilatation and Curettage			
	Drainage of Bartholin's abscess / cyst			
	Tubal ligation			
	FNAB of breast lump			
	Insertion of IUCD			
	Papanicolau (cervical) smears			
	Culdocentesis			
	Hormone implants			
	Laparotomy for ectopic pregnancy			
	TOP (if no religious/ethical objections)			
**Clinical governance**	MOU support, the perinatal audit meetings and PPIP programme, the training and audits of the basic antenatal care and perinatal education programmes and intrapartum audits			

Paediatrics

Clinical topic	Clinical skills – aim is D for unshaded skills and C for shaded skills	Numbers done (<5, 5-10, >10)	Grade the registrar 1st assess,2nd assess	
**Child**	Assess growth and classify malnutrition			
	Assess child abuse (sexual/non-sexual)<br>Assess child abuse (sexual/non-sexual)			
	Capillary blood sampling - finger, heel			
	CXR in a child			
	Developmental assessment			
	How to do and interpret Tine test and Mantoux tests			
	Intra-osseous line			
	IV access in a child			
	Lumbar puncture			
	Suprapubic bladder puncture			
	Venepuncture - upper limb, extn jugular vein			

Anaesthetics

Clinical topic	Clinical skills – aim is D for unshaded skills and C for shaded skills	Numbers done (<5, 5-10, >10)	Grade the registrar 1st assess,2nd assess	
**Anaesthetics**	Ring block			
	Administer oxygen			
	Check Boyle's machine			
	Control airway – mask and ambu bag			
	General anaesthetic			
	Inhalation induction			
	Intravenous induction			
	Intubate & ventilate patient			
	Ketamine anesthesia			
	Monitor patient during anaesthetic			
	Recover patient in recovery room			
	Reverse muscle relaxation (mix drugs)			
	Set airflows – Magill, Circle, T-piece			
	Spinal anaesthetic			
	Sterilize your equipment			
	Bier's block			
	Brachial block			

	Conscious sedation – basic			
	Epidural			

Surgery

Clinical topic	Clinical skills – aim is D for unshaded skills and C for shaded skills	Numbers done (<5, 5-10, >10)	Grade the registrar 1st assess,2nd assess	
**Adult health - general**	Wound care and dressings			
	Lymph node excision biopsy			
**Adults-Abdomen**	I&D of perianal haematoma			
	Proctoscopy			
	Appendicectomy			
	Interpret barium swallows			
**Adults-Urology**	Penile block			
	Reduce a paraphimosis			
	Circumcision			
	Drain hydrocoele			
	Insert a urinary and suprapubic catheter			
	Hydrocoelectomy			
	Interpret IVP for renal colic			
	Vasectomy			
	Orchidectomy and anchoring of torted testis			
**Skin**	Skin graft			
**Emergency**	Debride wounds or burns			

	I&D abscesses			
	Laparotomy for initial damage control in stabbed abdomen			

Orthopaedics

**Clinical topic**	**Clinical skills – aim is D for unshaded skills and C for shaded skills**	**Numbers done (<5, 5-10, >10)**	**Grade the registrar 1st assess,2nd assess**	
**Orthopaedics**	Measure shortening of the legs			
	Aspirate and inject the knee			
	Inject tennis elbow / golfers elbow			
	Inject the shoulder (ACJ, subacromial, GHJ)			
	Inject trochanteric bursitis			
	Interpret x-rays of joints			
	Apply finger and hand splints			
	Apply POP (upper and lower limbs)			
	Closed reductions (hand, forearm,tib-fib)			
	Set up traction (skeletal and skin)			
	Reduce elbow dislocation			
	Reduce hip dislocation			

	Reduce shoulder dislocation			
	Reduce radial head dislocation			
	Excise a ganglion			
	Inject carpal tunnel syndrome			
	Inject de Quervains tenosynovitis			
	Amputations-fingers/toes and lower limb			
	Apply club foot POP			
	Debridement of open fractures			
	Fasciotomy			

Emergencies

Clinical topic	Clinical skills – aim is D for unshaded skills and C for shaded skills	Numbers done (<5, 5-10, >10)	Grade the registrar 1st assess,2nd assess	
**Emergency**	CPR adult advanced support			
	CPR child advanced support			
	Choking			
	Primary survey			
	Intubate and manage airway			

	Cricothyroidotomy			
	Give oxygen			
	Insert chest drain			
	Relieve tension pneumothorax			
	IV cutdown			
	Secondary survey			
	Measure the GCS			
	Insert NGT			
	Interpret x-rays in trauma			
	Immoblise spine			
	Transport critically ill			
	Remove a splinter, fish-hook			
	Suture lacerations			
	Give a blood transfusion			
	Gastric lavage			
	Manage snake bite			
	Administer rabies prophylaxis			
	Selecting emergency equipment for doctors bag or emergency tray			
	Calculate % burnt			

	Certifying patient under mental health care act			
	Relieve cardiac tamponade			
	Peritoneal lavage			
	Suturing lip with tissue loss from human bite			
	Tracheostomy			

Communication

**Clinical topic**	**Clinical skills – aim is D for unshaded skills and C for shaded skills**	**Numbers done (<5, 5-10, >10)**	**Grade the registrar 1st assess,2nd assess**	
**Consultation**	Patient-centred consultation (all ages)			
	Holistic (3-stage) assessment and management			
	Motivate behaviour change			
	Break bad news			
	Counselling skills for HIV, TOP, after rape			
	Assess and consult couples, families			
	Conduct a family conference			
	Mini mental examination			
	Support / consult with PHC nurse			

	Use genogram and ecomap			
	Use problem-orientated medical record			
	Develop and use flowcharts for chronic care			
	Cope with language barriers			

ENT, Eyes, Skin and Miscellaneous

**Clinical topic**	**Clinical skills – aim is D for unshaded skills and C for shaded skills**	**Numbers done (<5, 5-10, >10)**	**Grade the registrar 1st assess,2nd assess**	
**ENT**	Remove a foreign body from the ear			
	Remove a foreign body from the nose<br>Syringe, dry swab an ear			
	Take a throat swab			
	Manage epistaxis (cautery, packing)			
	Assess hearing loss			
	Suture a pinna, lobe			
	Drain a peritonsillar abscess			
	Tonsillectomy / adenoidectomy			
	Reduce a fractured nose			
	Interpret audiogram			

**Skin**	Skin patch testing			
	Excise sebaceous cyst (other lumps-bumps)			
	Skin biopsy (punch and fusiform), skin scrapes			
	Wide Needle Aspiration Biopsy lymph node in HIV			
	Cryotherapy/cauterization			
	Phenol ablation of ingrown toenail			
	Inject keloids			
**Admin**	Work assessment and DG forms			
	Making appropriate referrals and letters			
	Completing sick certificates			
	Completing death certificates			
	Manage a clinic for chronic care (e.g. HIV, diabetes)			
**Forensic**	Completing J88			
	Assess, manage and document sexual assault			
	Assess, manage and document drunken driving			
	Assess, manage and document interpersonal violence			

**Palliative care**	Counselling of dying patient			
	Hypodermoclysis (subcutaneous infusion)			
	Set up a syringe driver			
**Eyes**	Fundoscopy (diabetes, hypertension), visual fields, visual acuity			
	Instill drops or apply ointment			
	Remove a foreign body in the eye, eversion of eyelid			
	I&D a chalazion			
	Suture an eyelid			
	Test for squint			
	Washout of eye (chemical burns)			
	Subconjunctival injections			
	Use a Schiotz tonometer			

Date completed:	
Comments on the registrar’s competency or professionalism	
Name of supervisor	Signature supervisor
Signature registrar	

Date completed:	
Comments on the registrar’s competency or professionalism	
Name of supervisor	Signature supervisor
Signature registrar	

Date completed:	
Comments on the registrar’s competency or professionalism	
Name of supervisor	Signature supervisor
Signature registrar	

Date completed:	
Comments on the registrar’s competency or professionalism	
Name of supervisor	Signature supervisor
Signature registrar	

SECTION 10 (optional)

Addendum / Lists

CUMULATIVE RECORD OF ROTATIONS / ATTACHMENTS

Start and End Dates of Rotation	No. of Months	Type of exposure / rotation

POST-GRADUATE LECTURES, MEETINGS, WORKSHOPS, SEMINARS, SYMPOSIA, CONGRESSES

Attendance at, or own presentations, at post-graduate meetings, lectures, workshops, symposia or congresses relevant to Family Medicine

(Attach *Certificates of Attendance* if applicable)

**Date**	**Duration**	**Presenter** (Self/other)	**Topic**	**Event**

CERTIFICATES of Courses relating to Family Medicine

(Copies of Certificates *must* be attached)

COURSE	INSTITUTION	DATE	COURSE DIRECTOR

ANY OTHER LEARNING EXPERIENCE RELEVANT TO FAMILY MEDICINE, that has not been captured, e.g. journal article publications:

……………………………………………………………………………………………

……………………………………………………………………………………………

……………………………………………………………………………………………

……………………………………………………………………………………………

……………………………………………………………………………………………

……………………………………………………………………………………………

……………………………………………………………………………………………

……………………………………………………………………………………………

……………………………………………………………………………………………

……………………………………………………………………………………………

SECTION 11

End of Year Assessment of Portfolio

Year 1

The portfolio is: Poor Barely adequate Average Good Excellent

Organization: Good Could be better Disorganized

Content: Good evidence of learning? Poor evidence of Learning?

Recommendations:

____

Signed:________________________________

HOD name:_____________________________

Date:__________________________________

Year 2

The portfolio is: Poor Barely adequate Average Good Excellent

Organization: Good Could be better Disorganized

Content: Good evidence of learning? Poor evidence of Learning?

Recommendations:

____

Signed:________________________________

HOD name:_____________________________

Date:__________________________________

Year 3

The portfolio is: Poor Barely adequate Average Good Excellent

Organization: Good Could be better Disorganized

Content: Good evidence of learning? Poor evidence of Learning?

Recommendations:

____

Signed:_________________________________

HOD name:______________________________

Date:___________________________________

Final

The portfolio is: Poor Barely adequate Average Good Excellent

Organization: Good Could be better Disorganized

Content: Good evidence of learning? Poor evidence of Learning?

Recommendations:

____

Signed:_________________________________

HOD name:______________________________

Date:___________________________________

SECTION 12

DECLARATION OF COMPLETION OF PORTFOLIO

To be completed at the END of 3 years, prior to final submission for the FCFP exams.

I, …………………………………………………………….hereby do solemnly declare that all information contained in this PORTFOLIO OF LEARNING is a true and accurate record of my professional experience, education and training from ………………. to ……………………… representing the period of training for the FCFP and MMed(Fam Meds) qualification.

Signature of Registrar: ....

Name of Registrar:..

Trainee Number:...

Date: ....
