# Supplementary material for: Development of a portfolio of learning for postgraduate family medicine training in South Africa: a Delphi study
Source: BMC Fam Pract. 2012 Mar 3;13:11. doi: 10.1186/1471-2296-13-11 (PMC3317832; doi:10.1186/1471-2296-13-11)
Supplement: Additional file 3 — Portfolio Guide version 1. [file 1471-2296-13-11-S3.PDF]

CMSA

Guide to the

PORTFOLIO OF LEARNING

Towards the Fellowship of the College of Family Physicians of South Africa

FCFP(SA)

AND

Master of Medicine in Family Medicine

MMed (Fam Med)

Dear Supervisor and Registrar,

Please take some time to study the *Guide to the Portfolio of Learning*, which should help you, together with your registrar, to complete a useful, reliable and valid learning portfolio.

As a supervisor, you have a commitment to one or more registrars for the period under your supervision. During this time, please plan to meet regularly to discuss the learning and development of your registrar. A Colleges of Medicine of South Africa (CMSA) workshop on assessment during November 2010 indicated 2 key issues:

- Transfer of theoretical knowledge into clinical practice is a big challenge.
- Registrars want and need feedback on their clinical practice in order to learn.

The portfolio should be the vehicle that facilitates these learning conversations or educational meetings. However, the workshop also highlighted the importance of the *people* using the portfolio (and various assessment tools). The portfolio per se is a tool, and its quality is determined by the quality of the supervision, the feedback, the context of learning, and the input from the registrar. The portfolio must not be a (thick) paper exercise, but rather a (lean) way of showing key evidence of learning; indicating continuous reflection on clinical practice and regular interaction between registrars and supervisors.

From 2013 onwards all registrars will need to sit a single exit exam offered by the CMSA. One requirement for entrance to the Part 1 exam will be an acceptable portfolio of learning. This implies that all new registrars starting in 2011 must start to develop such a portfolio. Therefore in 2011 all the Divisions / Departments of Family Medicine in South Africa have incorporated the learning portfolio into their assessment of training in the MMed (Family Medicine) programme.

The portfolio will be assessed by the academic head of department at the relevant university at the end of every year, for 3 years, as part of a summative assessment process (year mark). A recommendation (satisfactory / not satisfactory) will be given to the CMSA in the registrar's 4th year of training, 3 months prior to applying for the Part I examination, as a pre-requisite to sit the FCFP/MMed examination.

During a national Delphi consensus process, with experts and supervisors in Family Medicine in 2010, consensus was reached on 50 out of 85 learning outcomes which will be assessed by the Learning Portfolio. Simultaneously with this, there was a process to revise the national learning outcomes, which were previously reviewed in 2004. The Delphi process also asked panel members which assessment methods and tools would be the most appropriate to use in the portfolio. A 3-hour focus group discussion between the 8 national Family Medicine Head of Departments during late 2010 verified and clarified the new national outcomes, as well as agreeing on the final assessment methods as suggested by the Delphi panel members.

A large margin of flexibility and local adaptability for each university is accepted, while the general template of the portfolio, including the agreed upon national training outcomes, are standardised for South Africa as a whole.

This guide (and the accompanying portfolio) contains the following to assist you:

1. National Family Medicine 5 unit standards and 50 outcomes to be assessed in the portfolio.
2. Learning Plan
3. Assessment Methods and Tools.
4. Learning Conversations / Educational Meetings.
5. References

National learning outcomes to be assessed in the portfolio

Please look at the summary of the 50 national learning outcomes in section 3 of the Portfolio. To remind the registrar and supervisor of what has been covered and what still needs to be done and to plan appropriately, it is suggested that the registrar marks off what has been completed using the blocks in the "frequency" column (e.g. with an X or a tick or a number, or even colour the block in) during the course of registrar training.

Preparing a Learning Plan

You must meet with your local supervisor at the beginning and end of every rotation, or every 6 months (twice a year) if you are not working in formal rotations (for example at a district hospital for the whole year), to develop, document and review your learning plan. You must list the learning objectives you have set for yourself for the duration of that rotation or 6-month period. These could be updated as your rotation progresses.

On completion of the rotation, you must reflect on the progress you made in meeting your objectives, and identify areas in which further learning is needed.

Note that this is not an assessment by the supervisor of the registrar's work during the rotation. It is an exploration of the registrar's ***insight*** into the learning appropriate to that rotation and the extent to which it has been achieved.

The Learning Plan includes the following objectives:

- Identification of prior learning
- Identification of current learning needs (objectives)
- Planning of activities to meet these needs
- Timelines and support required to enable these activities to take place
- How learning will be evaluated (with the suggested tools)

The learner needs to be able to adjust their learning plan with each rotation and as they progress in the programme as a whole in order to develop the skill of lifelong learning and personal growth. Learning is best when it is learner-centred and very individual!

You need to keep in mind:

1. The National training outcomes for Family Medicine in SA.
2. Your University's MMed curriculum and its outcomes.
3. Your personal learning needs.
4. The focus of your planned rotations on the health service platform.

For example when you develop your learning plan you may need to simultaneously consider what you will be doing in your academic programme (e.g. modules, assignments), what practical experience you will be receiving in your clinical setting (e.g. your rotations) and what your personal learning needs are. Ultimately all of this must contribute towards achieving the outcomes of the programme and your own personal growth.

Linking to your Staff Performance Management System

Most provincial Departments of Health have a system of staff performance and development in place. Although registrars may be excluded from this workplace-based assessment, some provinces may still require it. If this is the case, then your Learning Plan should also be linked to your annual Staff Performance and Development Plan.

Assessment Methods and Tools

Different assessment methods and tools are available in the literature and used by different Departments of Family Medicine. The most commonly used methods and tools are summarized below, and some examples are given. The portfolio allows for various tools to be used (and shared) by different medical schools.

The 'bottom line' for whatever method or tool is used is that it provides clear evidence of learning for one of the expected outcomes. Your university will already have a number of assessment tools in place to monitor your development as a registrar. Make use of whatever relevant methods or tools you have in your programme and add them to your portfolio. Whenever possible therefore you should use existing assessment methods and tools in order to complete your portfolio. For example, if you are doing a relevant written assignment (e.g. COPC project, patient study, practice audit) as part of your academic programme, you should include this, together with the assessment scores you received, in your portfolio.

Written assignments:

Written assignments may be used to provide evidence of learning in any of the following areas (see also the table on outcomes and assessment methods in the portfolio):

1. Clinical competence (e.g. patient studies that demonstrate diagnostic reasoning, bio-psycho-social approach)
2. Family-orientated Primary Care
3. Ethical reasoning and medico-legal issues
4. Community-orientated Primary Care
5. Clinical governance

a. Evidence-based Medicine (e.g. critical appraisal of a journal article, searching for evidence, use of guidelines)
b. Quality improvement cycle / audit
c. Significant event analysis (SEA)
d. Morbidity and mortality meetings
e. Monitoring and evaluation meetings

One written assignment may show evidence of learning for more than one outcome. The registrar will do well to take note of this in his/her reflections, and indicate this overlap.

Observation by supervisor:

The immediate or overall supervisor must directly or indirectly (by use of audio or video tapes) observe the registrar during patient consultations, teaching events (where the registrar teaches or trains others), and when performing procedural or clinical skills. The following tools, which are also shown below, are useful here:

1. Mini-Clinical Evaluation Exercise (Mini-CEX) tool
2. Observed consultation and Clinical Question Analysis (CQA) tool
3. Communication skills observation tool
4. Significant event analysis

To optimise registrar-supervisor interaction and efficient use of educational time together, one tool could assess learning in a number of different outcomes.

Multi-source feedback

To assess ability to work in a team (as team member or leader) the following tools are useful:

1. 360 degrees questionnaire
2. Peer review

Log book review

Every university programme has its own registrar logbook. The logbook is just one assessment method within the portfolio. It primarily captures the number of clinical skills performed and the competence achieved.

A list of clinical skills that should be assessed in the logbook is included in the portfolio and based on the agreed national list of clinical skills for Family Medicine. You can use this logbook, or prefer to use your university programme's logbook.

How should the registrar be assessed via these assessment tools?

Every item that is entered into the portfolio should be assessed in some way or another, by a supervisor or a lecturer in the academic programme. This will assist the end-of-year overall assessment of the portfolio by the head of department.

The general recommendation by the national panel of experts and supervisors is to use one of two grading methods:

- A Global Rating (e.g. not satisfactory / needs improvement / satisfactory) for the item
- A specific Grade (e.g. percentage).

Many university academic programmes already give a mark for various assignments, which should just be captured in the portfolio, without the need for repeat assessment.

Educational meetings

A useful resource was published in the SA Family Practice Journal in 2010, discussion various learning conversations. The abstract and reference is:

Mash R, Goedhuys J, D'Argent F. Enhancing the educational interaction in family medicine registrar training in the clinical context SA Fam Pract 2010;52(1):51-54: "The relationship between registrar and trainer functions best when the trainer consciously facilitates the registrar's learning and considers all their interactions as educational opportunities. The trainer's role is more that of an educational guide and less that of an authoritarian expert. Both the registrar and the trainer should be aware of their own learning styles and how these may be complementary or contradictory. A variety of conversations with different purposes should be structured and planned and not left to chance and a number of methods for observing and collecting the registrar's clinical experience should be developed and used regularly. Further attention needs to be paid to the development of useful, reliable and valid portfolios."

During the programme the registrar should meet individually with their immediate supervisor and as a group of local registrars. These meetings can be alternated weekly (i.e. one week with your supervisor one-on-one and the next week as a group) and should last 1-2 hours and be recorded in the portfolio. The meetings could focus on one of the following learning opportunities:

A: Setting a learning agenda (at the beginning and end of a rotation or every 6-months): Reflection on the registrars experience to date, expectations or progress and planning of learning activities and goals for the next period.

B: Intermittent evaluation: For the registrar and trainer to check progress, review the portfolio, discuss any difficulties in their relationship or the organization that impede learning or service delivery, make new plans. Feedback can also be given and received on the programme or registrars performance.

C: Clinical / communication skills: Observation/audio/video-review of communication, consultation or procedural skills and feedback with role-play or simulation. Other clinical skills might also be demonstrated.

D: Case discussions: Reflect on your actual patients through the use of record review, presentation of problem patients or clinical tutorials on specific topics. Reflect on

difficult consultations, emotions or ethical dilemmas that arise from your clinical practice or setting.

E: Evidence based practice: Reflect on and critically appraise current journals and original research.

F: Other: For example co-ordination of on-line learning tasks with the on-site professional experience and service priorities i.e. topic for the quality improvement cycle.

The portfolio at the end of the year should demonstrate engagement with all of the above learning conversations and a minimum of 2-hours of educational meetings per month / 24-hours for the year. This is a minimum standard and in normal circumstances the portfolio should show engagement well above this.

Useful references and resources:

1. Instruments for Workplace-based Assessment (WBA): Follow link from: www.fdg.unimaas.nl/educ/cees/sa
2. Govaerts MJB, Van der Vleuten CPM, et at. Broadening Perspectives on Clinical Performance Assessment: Rethinking the nature of In-training Assessment. Advances in Health Sciences Education 2007; 12:239-260
3. Couper I, Mash B. Obtaining consensus on core clinical skills for training in family medicine SA Fam Pract 2008;50(6):69-73
4. Mash R, Goedhuys J, D'Argent F. Enhancing the educational interaction in family medicine registrar training in the clinical context SA Fam Pract 2010;52(1):51-54

The following section gives specific examples of the tools mentioned above from various university departments.

COMMUNICATION SKILLS OBSERVATION TOOL

Registrar name………………………………. Supervisor ……………………….

**Checklist score**<br>Each of the items below is an important skill in the consultation and should be rated separately. Rating should be at the performance expected from a family physician.	**Shown**	**Partially shown / not sure**	**Not shown**
**Initiating the session**			
Makes appropriate greeting / introduction and demonstrates interest and respect<br><br>Greets patient, obtains name, introduces self, attends to physical comfort of patient, shows interest and respect, establishes initial rapport.			
Identifies and confirms the patient's problem list or issues<br><br>Gives an opportunity for the patient to list all their issues or problems before exploring the initial problem "So headache, fever - anything else you'd like to talk about?". Summarises and confirms the list with the patient.			
**Gathering information**			
Encourages patient's contribution / story<br><br>By use of open as well as closed questions, attentive listening, facilitation skills and summarization and responding to cues. As opposed to cutting off the patient, use of only closed questions in an interrogatory style.			
Makes an attempt to understand the patient's perspective<br><br>Elicits spontaneously and acknowledges the patient's perspective or uses specific questions– beliefs, concerns, expectations, and feelings.			
Thinks family, and obtains relevant family, social and occupational information<br><br>Elicits relevant information about the patient's household, family, occupation, and environment.			
Obtains sufficient information to ensure no serious condition is likely to be missed<br><br>Elicits enough clinical information to establish a working diagnosis and ensure no serious condition is likely to be missed.			
**Explanation and planning**			
Appears to make a clinically appropriate working diagnosis<br><br>The apparent diagnosis is clinically appropriate according to the subjective and objective evidence. If necessary the notes in the patients folder can be reviewed later to establish what the doctor was thinking.			
There is a clear explanation of the diagnosis and management plan			

The explanation is well organized, in small chunks, avoids jargon, where appropriate makes use of visual methods, leaflets, repetition, signposting.			
Gives patient an opportunity to ask for other information and / or seeks to confirm patient's understanding<br><br>The patient is asked if they would like other information and / or their understanding is checked by reverse summarizing or opportunity to clarify			
The explanation takes account of and relates to the patient's perspective<br><br>The explanation connects, responds to or takes into account the patient's beliefs, concerns and expectations			
Involves the patient where appropriate in decision making<br><br>The patient is offered insight into doctor's thought processes, suggestions, options and invited to participate in decision making through use of choice, expression of preferences or ideas. The doctor does not just give orders, directives or instructions of what must be done.			
Chooses an appropriate management plan<br><br>The management plan is based on scientifically sound evidence and is appropriate for the diagnosis. If necessary the notes in the patients folder can be reviewed later to establish what the doctor was thinking.			
**Closure**			
Closes consultation successfully in the time available<br><br>Brings the consultation to a conclusion rather than running out of time. Deals with any remaining issues from the patient.			
Provides appropriate safety netting for the patient<br><br>Shows evidence of having considered how certain they are of the diagnosis, what might go wrong with the treatment, how they will know if things do not go well, side effects occur or more serious sequelae develop. Shows this in an appropriate plan of safety netting with the patient.			
**Additional skills – for merit**<br><br>These will not be applicable to all consultations, but will depend on the content of the specific consultation			
Establishes therapeutic rapport / relationship in a patient with a mental or psychosocial problem<br><br>Shows evidence of basic counseling skills used in a mature and integrated way that offers supportive therapy to the patient: such as empathy, attentive listening, summarizing, unconditional positive regard, facilitative responses.			
Breaks bad news appropriately<br><br>Shows evidence of structured approach to breaking bad news that includes skills such as: setting the scene by summarizing or discovering where things have reached to date and check patients understanding; warn patient that difficult information is coming; give information clearly, directly and honestly; be sensitive to the emotional reaction from the patient by giving space for it, encourage expression of feelings; allow patient to ask their own questions, express concerns and elicit the type and amount of information they want, make a supportive plan.			

Shows skills in brief motivational interviewing<br><br>Shows evidence of brief motivational interviewing skills such as: setting an agenda, explores readiness to change, chooses skills appropriate to the patients readiness to change (elicit-provide-elicit, decision balance sheet, brainstorming), rolls with resistance.			
**Overall global rating**<br>Consider your overall impression of the consultation, including the doctor-patient relationship and the structure and flow of the session. **Select ONE of the boxes opposite:**	**At the level of a family physician**	**Not sure / borderline**	**Definitely not at the level of a family physician**

CLINICAL QUESTION ANALYSIS

This sheet should be with you during your practice and act as a guide to ask questions in a moment of reflection alone after the patient consultation. It can also be used to reflect on other challenges or situations that arise in clinical practice. The supervisor signs after discussing your notes here.

a. The Situation and/or Patient Actually Met Needs (PAN) at time of consultation

....

....

....

b. The Situational Difficulty and/or Patient Unmet Need (PUN) (on Reflection)

....

....

....

c. MY Problem, difficulty, questions or observations (including my emotional reactions on reflection)

....

....

………

d. MY (Doctor) Educational Need (DEN) (Which aspects of this encounter or situation do I need to find out more about to improve?)

………

………

………

e. How did I close the learning loop i.e. what did I do in my practice differently or implement what I learnt?

………

………

………

Registrar……………………………Signature………………………………Date…………………………

OBSERVED CONSULTATION

The Supervisor can use the Clinical Question Analysis Tool together with the Observed Consultation (OC) below. The Registrar should fill in the Clinical Question Analysis Tool after discussing the consultation with their supervisor. The Supervisor can also review later on how the learning loop was closed, i.e. what was learnt from the Doctors Educational Need and how practice changed.

<u>Notes:</u>

- Initially, while registrars are in training, detailed remediation should take place during the consultation; student should also be asked for his/her hypothesis after taking the history. As students progress, this intervention should decrease, until the consultation is purely observed, unless the patient's welfare is endangered.
- No intervention should take place during formal (summative) assessment.
- During formal summative assessment, registrars may need help to focus on specific issues in patients with complex problems, where time has been limited.
- Time management is an important skill, but registrars can be assessed out of what was appropriately completed, where there are clear reasons why the consultation could not be finished within the time allowed; assessors may intervene 1 minute before the end, or afterwards, to ask for the registrar's assessment and plan.

OBSERVED CONSULTATION ASSESSMENT (USE WITH Clinical Question Analysis)

Element	Details	Rating*	Comments
**Facilitation**	• Establishes rapport<br>• Communicates well (verbally/non-verbally)<br>• Responds to cues (verbal/non-verbal)<br>• Active, attentive listening<br>• Behaviour towards and relationship with patient<br>• Uses appropriate language		
**Clinical reasoning**	**S:**<br>• Takes a focused history, based on clinical reasoning<br>• History taking apparently based on clear hypotheses<br>• Appropriate, specific questions		
	**O:**<br>• Examines patient correctly and appropriately, apparently directed by hypotheses<br>• Demonstrates good clinical skills<br>• Performs appropriate side-room tests		
	**A_3:**<br>• Does a comprehensive bio-psycho-social assessment<br>• Evidence of considering all important possibilities (hypotheses)<br>• Bases assessment on findings (S&O) and clinical knowledge		
	**P_4:**<br>• Carries out all 4 tasks of the consultation as appropriate.<br>• Appropriate investigations/referral considered<br>• Plan is rational (based on S, O, A_3).<br>• Plan is comprehensive (investigations, observation, therapy/ medication, counselling, referral/ follow-up).		
**Collaboration**	• Explains and discusses findings (positive and negative).<br>• Involves patient in decision making.<br>• Negotiates plan with patient.		
**Integration**	• Logical, orderly process throughout consultation<br>• Appropriate management of time, within time constraints<br>• Patient centred approach<br>• Record keeping		
**TOTAL**	**Global score based on the above (rounded off to 5%)**	**/100**	
**Critical factors**	Student should fail if demonstrates unethical or unprofessional behaviour, dangerous practice, or clinical incompetence.		**PASS / BORDERLINE / FAIL**

**Registrar:**	**Name of Supervisor:**	**Signature:**

***Rating scale: E = Excellent (>75%); G = Good (60-75%); S = Satisfactory (50-59%); B = Borderline Fail (40-49%); F = Fail (<40%)**

MINI CLINICAL EVALUATION EXERCISE TOOL (MINI-CEX)

TEMPLATE FOR SKILLS ASSESSMENT

1. PREPARATION OF PATIENT

- Introduces self (if not already known)
- Puts patient at ease
- Explains the procedure to patient
- Explains indications, contraindications, risks and benefits of the procedure as applicable
- Appropriately answers any question(s) the patient might have
- Assures the patient that his/her comfort during the procedure is your priority
- Gets patient's consent

2. PREPARATION FOR PROCEDURE

- Uses appropriate safety measures
- Maintains sterility as required
- Prepares correct anaesthesia/analgesia
- Appropriate choice of needed material or instrument(s)
- Appropriate choice of needed drugs

3. PROCEDURE

- Places patient in correct position for the procedure
- Accurately and comprehensively performs the procedure
- Explains step by step what is being done
- Does not unnecessary hurt the patient
- Critical steps are not omitted

4. AFTER CARE

- Admits patient for observation if needed
- Arranges follow-up of patient
- Refers the patient when indicated
- Educates the patient about the condition
- Provides and/or prescribes analgesia, dressings, other appropriate management Provides preventive measures

5. TEAM WORK

- Works collegially with nursing or medical colleagues in performing the procedure
- Gives appropriate instructions to nursing staff involved
- Ensures proper hand over of patient for ongoing care if required

MINI CLINICAL EVALUATION EXERCISE - SKILLS

SUPERVISOR:______________________DATE:______________REGISTRAR: _____________________

SETTING: ☐ Ambulatory ☐ In-patient ☐ EC/Casualty Other: ______________

Patient age: _____ Patient sex: _____ ☐ New ☐ Follow-up

SKILL PERFORMED: ____

Complexity of skill:

Low ☐ Medium ☐ High ☐

---

1. PREPARATION OF PATIENT:

1 2 3 4 / 5 6 7 / 8 9 10

UNSATISFACTORY SATISFACTORY EXEMPLARY

---

2. PREPARATION OF EQUIPMENT:

1 2 3 4 / 5 6 7 / 8 9 10

UNSATISFACTORY SATISFACTORY EXEMPLARY

---

3. PROCEDURE:

1 2 3 4 / 5 6 7 / 8 9 10

UNSATISFACTORY SATISFACTORY EXEMPLARY

---

4. AFTER CARE:

1 2 3 4 / 5 6 7 / 8 9 10

UNSATISFACTORY SATISFACTORY EXEMPLARY

---

5. TEAM WORK:

1 2 3 4 / 5 6 7 / 8 9 10

UNSATISFACTORY SATISFACTORY EXEMPLARY

TOTAL **/50**

Comments:

……

……

……

Supervisor……………………….................……Signature…..........................……………Date……….......................

SIGNIFICANT EVENT ANALYSIS

Description of occurrence Date

What was managed well?

What did not go well i.e. briefly the identified problem?

Fishbone (put what you see as causes to this problem as the bones to the arrow pointing to the identified problem)

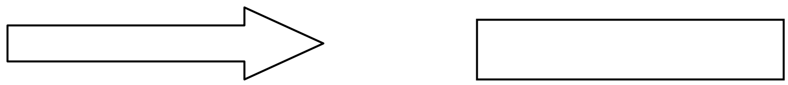

Identification of main learning needs
Important points from discussion
Conclusion of reflection
How could this have been better managed?
Suggestions, recommendations

Actions	By whom	When

Registrar.................................Supervisor…………………………..Signature……………………………..Date…………....
